# Evidence on physical activity and falls prevention for people aged 65+ years: systematic review to inform the WHO guidelines on physical activity and sedentary behaviour

**DOI:** 10.1186/s12966-020-01041-3

**Published:** 2020-11-26

**Authors:** Catherine Sherrington, Nicola Fairhall, Wing Kwok, Geraldine Wallbank, Anne Tiedemann, Zoe A. Michaleff, Christopher A. C. M. Ng, Adrian Bauman

**Affiliations:** 1 Institute for Musculoskeletal Health, The University of Sydney and Sydney Local Health District, Sydney, Australia; 2 School of Public Health, Faculty of Medicine and Health, The University of Sydney, Sydney, Australia; 3 Institute for Evidence-Based Healthcare, Faculty of Health Sciences and Medicine, Bond University, Gold Coast, Queensland Australia; 4 Medical School, Faculty of Health and Medical Sciences, The University of Western Australia, Perth, Australia; 5 Charles Perkins Centre, Prevention Research Collaboration, Sydney School of Public Health, Faculty of Medicine and Health, The University of Sydney, Sydney, Australia

**Keywords:** Accidental falls, Aged, Exercise, Older adults, Physical activity

## Abstract

**Background:**

Exercise prevents falls in older adults. Regular updates of estimated effects of exercise on falls are warranted given the number of new trials, the increasing number of older people globally and the major consequences of falls and fall-related injuries.

**Methods:**

This update of a 2019 Cochrane Review was undertaken to inform the World Health Organization guidelines on physical activity and sedentary behaviour. Searches were conducted in six databases. We included randomised controlled trials evaluating effects of any form of physical activity as a single intervention on falls in people aged 60+ years living in the community. Analyses explored dose-response relationships. The certainty of the evidence was assessed using Grading of Recommendations Assessment, Development and Evaluation (GRADE).

**Results:**

This review included 116 studies, involving 25,160 participants; nine new studies since the 2019 Cochrane Review. Exercise reduces the rate of falls by 23% (pooled rate ratio (RaR) 0.77, 95% confidence interval (CI) 0.71 to 0.83, 64 studies, high certainty evidence). Subgroup analysis showed variation in effects of different types of exercise (*p* < 0.01). Rate of falls compared with control is reduced by 24% from balance and functional exercises (RaR 0.76, 95% CI 0.70 to 0.82, 39 studies, high certainty evidence), 28% from programs involving multiple types of exercise (commonly balance and functional exercises plus resistance exercises, RaR 0.72, 95% CI 0.56 to 0.93, 15 studies, moderate certainty evidence) and 23% from Tai Chi (RaR 0.77, 95% CI 0.61 to 0.97, 9 studies, moderate certainty evidence). The effects of programs that primarily involve resistance training, dance or walking remain uncertain. Interventions with a total weekly dose of 3+ h that included balance and functional exercises were particularly effective with a 42% reduction in rate of falls compared to control (Incidence Rate Ratio (IRR) 0.58, 95% CI 0.45 to 0.76). Subgroup analyses showed no evidence of a difference in the effect on falls on the basis of participant age over 75 years, risk of falls as a trial inclusion criterion, individual versus group exercise, or whether a health professional delivered the intervention.

**Conclusions:**

Given the strength of this evidence, effective exercise programs should now be implemented at scale.

**Supplementary Information:**

**Supplementary information** accompanies this paper at 10.1186/s12966-020-01041-3.

## Background

One in three community-dwelling people aged over 65 years fall each year [1, 2] with the rate of fall-related injuries increasing with age [3]. Consequences of falls include fractures and head injuries [3], reduced quality of life [4], fear of falling, loss of confidence, and self-restricted activity levels leading to a reduction in physical function and social interactions [5]. In turn, the restriction of activities impedes physical capacity and exacerbates the risk of further falls.

Physical activity is as any bodily movement that requires energy expenditure and includes exercise (planned, structured and repetitive activity, and aims to improve or maintain one or more component of physical fitness) and leisure or lifestyle activities (e.g. walking, gardening). Exercise, as a single intervention, is the most commonly tested fall prevention intervention and a previous Cochrane Review showed exercise prevents falls [6]. Economic evaluations accompanying randomised trials have found exercise to be a cost-effective falls-prevention strategy [7]. Exercise interventions are effective when delivered in a group-based setting or on an individual basis. Multicomponent programs that target both strength and balance [6] and programs that include balance training appear to be particularly effective [8].

Regular updates of the estimated effects of exercise interventions on falls are warranted given the number of new trials published. The large numbers of older people globally and the long-term consequences of falls and fall-related injuries for individuals and health systems make it particularly important that the latest research is summarised regularly to confirm or modify conclusions from previous reviews. Different exercise programs may have different effects on falls and so careful analysis of the characteristics and impact of different programs is important [9].

This systematic review was undertaken to inform the World Health Organization guidelines on Physical Activity and Sedentary Behaviour [10] and involved an update of the Cochrane Review of randomised controlled trials published in 2019 [11] that found high certainty evidence that exercise interventions reduced the rate of falls by 23% in community-dwelling older people compared with controls (rate ratio (RaR) 0.77, 95% confidence interval (CI) 0.71 to 0.83; 12,981 participants, 59 studies). This update focuses on the review’s primary outcome, rate of falls.

## Methods

### Eligibility criteria

We included randomised controlled trials (RCTs), both individually and cluster randomised, evaluating the effects of physical activity interventions on falls in older people living in the community. To be included in the review, studies had to meet the following criteria: 1) Population: community-dwellers aged 60 years and older. Studies that included younger participants were included if the mean age minus one standard deviation was more than 60 years. Studies that included participants who were living in places of residence that provide residential health-related care or rehabilitation were excluded. Studies that only included participants with health conditions that increase the risk of falls, such as stroke, Parkinson’s disease, multiple sclerosis, dementia, previous hip fracture and severe visual impairment, were excluded. Several of these topic areas are covered by other Cochrane Reviews [12, 13]. We acknowledge that some individuals with these (and other) health conditions may be included in studies of the general community which we included; 2) Intervention: any physical activity interventions tested in trials where physical activity was a single intervention rather than a component of a broader intervention. We considered trials where an additional low-contact intervention (e.g. information on fall prevention) was given to one or both groups if we judged that the main purpose of the study was to investigate the role of exercise; 3) Outcome: falls with studies reporting the rate of falls (falls per person-year) being pooled for meta-analysis.

### Information sources and search

The present report updates the searches performed in the 2019 Cochrane Review [11], without deviation from its protocol and extending to studies published up to 7 November 2019. We searched: the Cochrane Bone, Joint and Muscle Trauma Group Specialised Register (2 May 2018 to 7 November 2019); the Cochrane Central Register of Controlled Trials. (CENTRAL) (Cochrane Register of Studies Online) (2018 Issue 1 to 7 November 2019); MEDLINE (including Epub Ahead of Print, In-Process & Other Non-Indexed Citations and MEDLINE Daily) (start 2018 to 7 November 2019); Embase (start 2018 to 7 November 2019); the Cumulative Index to Nursing and Allied Health Literature (CINAHL) (May 2018 to 7 November 2019); and the Physiotherapy Evidence Database (PEDro) (2018 to 2019), using tailored search strategies. We did not apply any language restrictions. In MEDLINE, we combined subject-specific search terms with the sensitivity- and precision-maximising version of the Cochrane Highly Sensitive Search Strategy for identifying randomised trials [14]. We also identified ongoing and recently completed trials by searching the World Health Organization International Clinical Trials Registry Platform (WHO ICTRP) and ClinicalTrials.gov (November 2019). The search strategies for the electronic bibliographical databases and trial registers are shown in Additional file 1.

### Study selection

For the updated search, two reviewers (NF, WK) independently screened the title, abstract and descriptors of identified studies for possible inclusion. From the full text, these review authors independently assessed potentially eligible trials for inclusion. Disagreements were resolved through discussion with a third author. We contacted trial authors for additional information as necessary.

### Data collection

Pairs of reviewers (CS, NF, WK) independently completed a pretested data extraction form (based on the one used in the Cochrane Review [15]). Disagreement was resolved by consensus or third party adjudication. Review authors were not blinded to the journal or authors names. Review authors did not assess their own trials. Full details of data extracted (excluding the nine new trials included in this update) are shown in Sherrington 2019 [11]. We used the Prevention of Falls Network Europe (ProFaNE) taxonomy to group similar physical activity interventions [16]. The ProFaNE category gait, balance, co-ordination, or functional task training was referred to as balance and functional exercises for simplicity. Full details and illustrative examples are shown in Additional File 2.

### Risk of bias and certainty of evidence

One review author (NF) assessed risk of bias using Cochrane’s Risk of bias tool as described in the Cochrane Handbook [17]. We constructed and visually inspected funnel plots. Using the Grading of Recommendations Assessment, Development and Evaluation (GRADE) framework [18], we assessed the certainty of the evidence as ‘high’, ‘moderate’, ‘low’ or ‘very low’ depending on the presence and extent of five factors: risk of bias; inconsistency of effect; indirectness; imprecision; and publication bias. We used standardised qualitative statements to describe the different combinations of effect size and the certainty of evidence [19].

### Synthesis of results

The treatment effects for rate of falls were reported as rate ratios (RaRs) with 95% confidence intervals (CIs). We assessed heterogeneity of treatment effects by visual inspection of forest plots and by using the Chi^2^ test (with a significance level at *P* < 0.10) and the I^2^ statistic. For our primary comparison, we pooled data from all relevant trials without stratification.

We performed subgroup analyses using Cochrane’s Review Manager 2014 [20] to compare the effect of physical activity on falls based on the following: trials that used an increased risk of falls as an inclusion criterion compared with those with general recruitment; trials with primarily older populations (defined by inclusion criteria 75 years or above, lower range limit more than 75 years, or mean age minus one standard deviation more than 75 years) compared with those with primarily younger populations; physical activity interventions delivered individually versus in a group setting; physical activity interventions delivered by health professionals versus trained fitness leaders and the different exercise intervention categories, according to ProFaNE taxonomy. We undertook sensitivity analysis to explore the findings regarding the impact of exercise coded as having multiple categories.

We undertook meta-regression using the user-written Stata command *metareg* [21] to explore the impact of hours of exercise intervention per week over the program period (hours of intervention are shown for each study in Additional file 5: Supplementary Tables 3 and Additional file 6: Supplementary Table 4) and used the *lincom* post-estimation command to estimate the impact of interventions that were of higher dose (3+ h per week) and that included exercises that target balance and function (i.e., balance and functional exercise, Tai Chi and multiple component exercise interventions that include balance and functional exercise). This analysis is an update of our previous non-Cochrane review of exercise on the rate of falls [8].

## Results

**Fig. 1:**
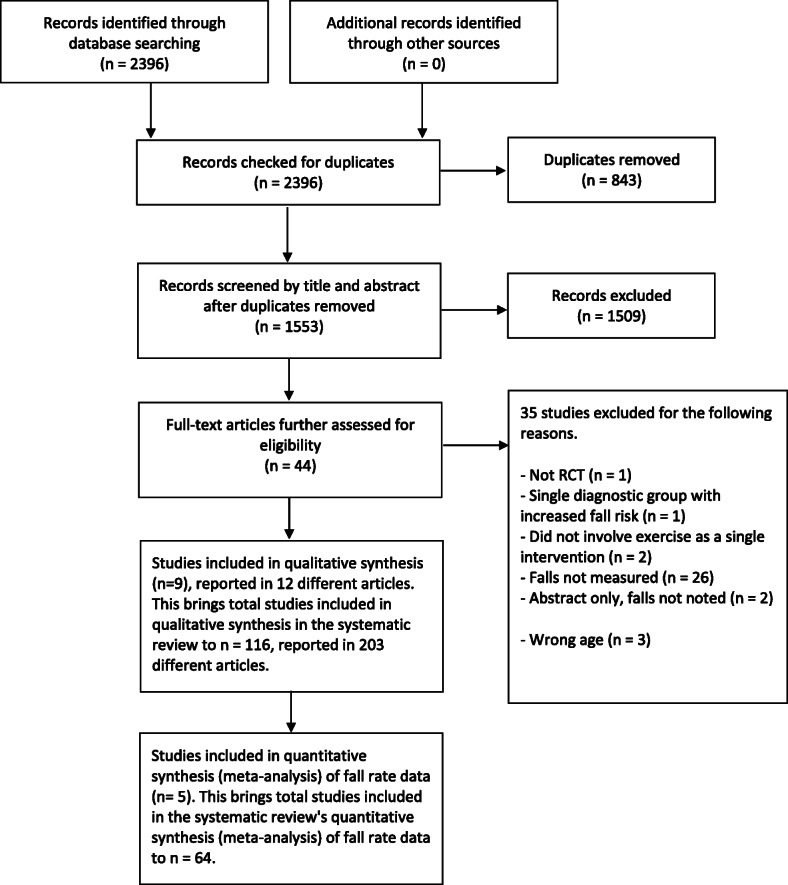
Flow of studies

Figure 1 shows the flow of records. In brief, the search update identified 2396 potentially eligible new records and the full text was screened for 44 studies. Study selection resulted in the inclusion of an additional nine studies not in the 2019 Cochrane Review and the exclusion of 35 studies. This update includes 116 studies; the 108 studies included in the 2019 Cochrane Review and the nine new studies, since one feasibility study [22] included in the 2019 Cochrane Review was replaced with the recently published full trial [23].

The characteristics and overall risk of bias of the 116 included trials are summarised in Table 1 and detailed in Additional File 3: Supplementary Table 1. The 116 included studies were all RCTs and involved 25,160 participants. Most of the trials were individually randomised and ten were cluster randomised. The included trials were carried out in 29 countries and three of the included trials were multinational trials. Overall, 74% of included participants were women. All participants were women in 28 trials and men in one trial. Sixty-four of these trials reported the rate of falls as an outcome. The duration of exercise intervention ranged from 2 weeks to 2.5 years and the total hours of exercise ranged from 4 to 1086 h.

**Table 1:** Summary of characteristics of 116 included studies

Characteristic	Overview of included trials, n (%) unless otherwise indicated
**Sample size, median (IQR)**	131 (66 to 249)
**Location**	
USA	21 (18%)
Australia	20 (17%)
Japan	11 (9%)
UK	7 (6%)
Canada	6 (5%)
Finland	5 (4%)
Brazil	4 (3%)
Germany	4 (3%)
New Zealand	4 (3%)
Sweden	4 (3%)
Other	27 (23%)
Multinational	3 (3%)
**Age, median (IQR)**	76 (72 to 80)
**Gender**	
Men: women, number (%) in included studies	6476 (26%): 18,684 (74%)
**Increased fall risk at enrolment**	62 (53%)
**Risk of bias, high risk**	
Sequence generation	0 (0%)
Allocation concealment	5 (4%)
Blinding of participants and personnel	6 (5%)
Blinding of outcome assessment	24 (21%)
Incomplete outcome data	33 (28%)
Selective outcome data	52 (45%)
Ascertainment bias	30 (26%)

*IQR* Interquartile range

Exercise (all types) reduces the rate of falls by 23% compared with control (RaR 0.77, 95% CI 0.71 to 0.83; 14,306 participants, 64 studies, I^2^ = 61%; high-certainty evidence). Subgroup analysis (Table 2) showed no evidence of a difference in the effect on falls for participants with an increased risk of falling as a trial inclusion criterion versus general recruitment, participants aged 75 years or above versus aged 60–74, group versus individual exercise, or whether interventions were delivered by a health professional versus a trained exercise leader. See Additional File 4, Supplementary Table 2: Summary of findings table: Rate of falls outcome (falls per person-years) for type of exercise for greater detail.

**Table 2:** Summary of results and subgroup analyses

Analysis/ GRADE evidence certainty rating	No. of participants / No. of Studies	Rate Ratio (95% CI)	I^**2**^	Test for sub-group difference
**Overall effect of exercise on rate of falls versus control**				
Exercise versus control/ high certainty	14,306 / 64	0.77 (0.71 to 0.83)	61%	-
**Subgroup analyses**				
**a) Based on type of exercise**^**a**^				
Balance, and functional exercises versus control/ high certainty	7989 / 39	0.76 (0.70 to 0.82)	31%	Chi^2^ = 18.91, df = 6, *P* = 0.004, I^2^ = 68%
Resistance exercises versus control/ very low certainty	327 / 5	1.14 (0.67 to 1.97)	67%	
Tai Chi exercise versus control/ moderate certainty	3196 / 9	0.77 (0.61 to 0.97)	83%	
Dance exercise versus control / very low certainty	522 / 1	1.34 (0.98 to 1.83)	–	
General physical activity (including walking) training versus control/ very low certainty	441 / 2	1.14 (0.66 to 1.97)	67%	
Multiple categories of exercise (often including, as primary interventions: gait, balance, and functional (task) training plus resistance training versus control/ moderate certainty	2283 / 15	0.72 (0.56 to 0.93)	65%	
**b) Based on fall risk at baseline**				
Increased risk of falling	7872 / 32	0.76 (0.69 to 0.84)	65%	Chi^2^ = 0.1, df = 1, *P* = 0.75, I^2^ = 65%
Not using increased risk of falling as entry criterion	6434 / 32	0.78 (0.68 to 0.89)	57%	
**c) Based on age**				
Aged ≥75 years	3841 / 14	0.85 (0.73 to 1.0)	61%	Chi^2^ = 2.29, df = 1, *P* = 0.13, I^2^ = 56%
Aged < 75 years	10,465 / 50	0.74 (0.68 to 0.81)	60%	
**d) Based on setting of the interventions delivered**				
Group	8909 / 43	0.74 (0.67 to 0.83)	66%	Chi^2^ = 1.3, df = 1, *P* = 0.31, I^2^ = 3%
Individual	5397 / 23	0.81 (0.72 to 0.91)	47%	
**e) Based on who delivered the intervention**				
Health professionals (usually physiotherapists)	5099 / 28	0.73 (0.64 to 0.82)	53%	Chi^2^ = 1.2, df = 1, *P* = 0.27, I^2^ = 16%
Non-health professionals (trained instructors)	9207 / 36	0.79 (0.72 to 0.88)	65%	

*CI* confidence interval

^a^Full details on classification of type of exercise and illustrative examples shown in Additional File 2

Subgroup analysis by exercise type showed a variation in the effects of the different types of exercise on rate of falls, the visual impression being confirmed by the statistically significant test for subgroup differences: Chi^2^ = 18.91, df = 6, *P* = 0.004, I^2^ = 68%. The rate of falls compared with control is reduced by 24% in balance and functional exercises (RaR 0.76, 95% CI 0.70 to 0.82; 7989 participants, 39 studies, I^2^ = 31%, high-certainty evidence), 23% in Tai Chi (RaR 0.77, 95% CI 0.61 to 0.97; 3169 participants, 9 studies, I^2^ = 83%; moderate-certainty evidence) and 28% in multiple types of exercise (commonly balance and functional exercises plus resistance exercises) (RaR 0.72, 95% CI 0.56 to 0.93; 2283 participants, 15 studies; I^2^ = 65%; moderate-certainty evidence). Table 2 shows the result for other types of exercise. The characteristics of studies in categories of exercise intervention that significantly reduced falls are described in Additional File 5: Supplementary Table 3. Sensitivity analyses revealed little difference in the results when we pooled only trials that include the most common two components (balance and functional exercises plus resistance exercises) (RaR 0.69, 95% CI 0.48 to 0.97; 1084 participants, 8 studies; I^2^ = 71%).

Compared with controls, we are uncertain whether the rate of falls is reduced in dance exercise (RaR 1.34, 95% CI 0.98 to 1.83; 522 participants, 1 study; very low-certainty evidence) and in walking programs (RaR 1.14, 95% CI 0.66 to 1.97; 441 participants, 2 studies; I^2^ = 67%; very low-certainty evidence). The characteristics of studies in categories of exercise not found to be effective in preventing falls are outlined in Additional File 6: Supplementary Table 4.

**Fig. 2:**
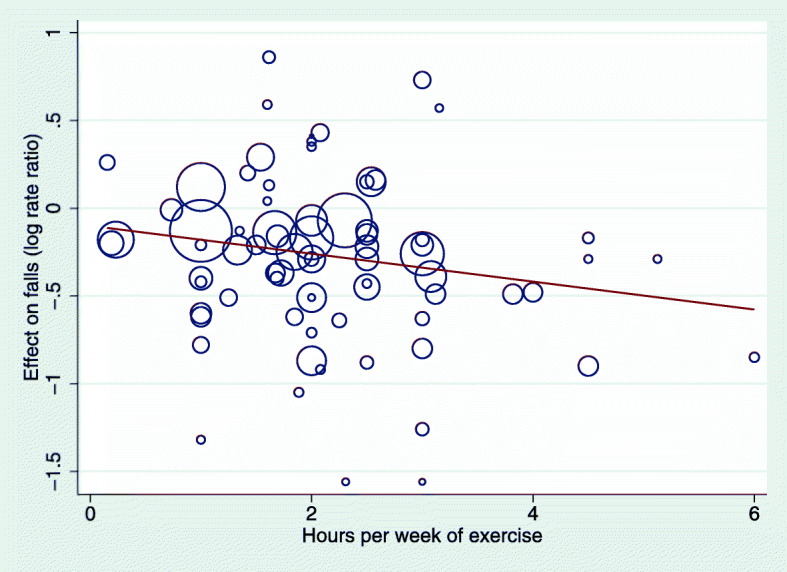
Relationship between effect of intervention on rate of falls (i.e., the between-group difference from each trial) and hours of exercise per week

Meta-regression analyses suggested a dose-response relationship. There was a greater reduction in falls from exercise programs that involved more hours per week (Fig. 2) although this did not reach statistical significance (*p* = 0.077). Interventions that included an exercise dose of more than 3 h per week and included balance and functional exercises were particularly effective with an estimated 42% reduction in the rate of falls (IRR 0.58, 95% CI 0.45 to 0.76). Table 3 shows the estimated impact of the separate and combined impact of dose and exercise type on falls.

**Table 3:** Estimated impact of dose and exercise type on falls

Feature	Effect on falls, IRR (95% CI)
Higher dose, 3+ h per week of total exercise	0.83 (0.60 to 1.15)
Inclusion of balance/ functional exercises^a^	0.76 (0.69 to 0.83)
Higher dose, 3+ h per week of total exercise plus inclusion of balance/ functional exercises^a^	0.58 (0.45 to 0.76)
Lower dose, < 3 h per week of total exercise and no inclusion of balance/ functional exercises^a^	1.08 (0.84 to 1.38)

^a^balance and functional exercise, Tai Chi and multiple component exercise interventions that include balance and functional exercise

### Heterogeneity and risk of bias across studies

**Fig. 3:**
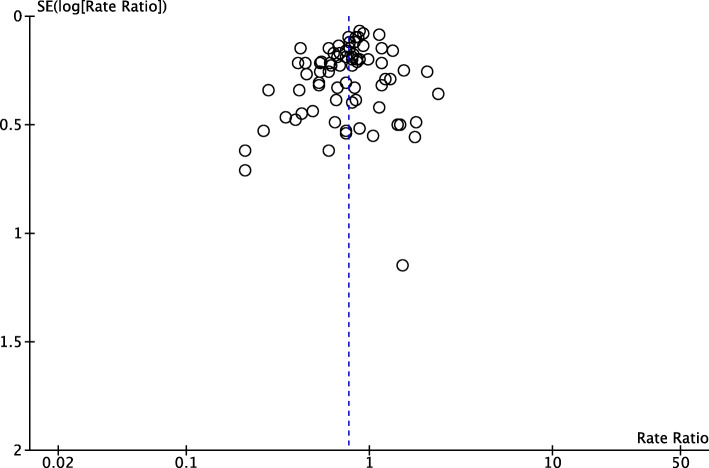
Funnel plot for the comparison of exercise versus control on rate of falls

This review’s analyses display minimal to substantial heterogeneity with *P* < 0.05 for the Chi^2^ test and I^2^ values up to 83%. This variability was not explained by our subgroup analyses. We consider this likely to represent between-study differences in the exact nature of programs (e.g. dose, intensity, adherence) and target populations, which requires ongoing investigation. Given the overall positive impact of the programs and the stability of results, we do not consider this to negate the findings of the meta-analyses we have undertaken. The funnel plots (Fig. 3) show some asymmetry but we did not consider the asymmetry sufficient to downgrade the level of evidence.

## Discussion

This updated review identified 116 studies and of these, 64 RCTs provided data on rate of falls that were suitable for pooling. These provide high-certainty evidence that exercise reduces the rate of falls in older adults living in the general community by approximately 25%. Although subgroup analyses did not reveal differences in effect on falls according to falls risk at baseline, there will be a greater reduction in the absolute numbers of falls in the higher risk population. Subgroup analyses did not reveal differences in the effect on falls according to whether trials included younger and older populations based on a 75 year cut-off, or whether interventions were delivered by a health professional versus trained instructors who were not health professionals. These findings suggest that exercise programs should be delivered to older or higher risk individuals as well as to the general community. Overall, programs delivered by health professionals are not more effective than programs delivered by trained instructors, although it is likely that the provision of exercise to high risk people can be more safely and effectively undertaken by health professionals. Subgroup analyses did not reveal differences in effect on falls according to whether interventions were delivered in a group setting or delivered individually. This suggests that either delivery mode can be effective and participants can choose whichever suits their preferences and other commitments, depending on the availability of programs and services.

There were significant subgroup differences for rate of falls when sub-grouped by exercise type. Exercise programs that primarily involve balance and functional exercises reduce falls. Tai Chi and programs that include multiple exercise categories (typically balance and functional exercises plus resistance exercises) probably reduce falls. We are uncertain of the effect of resistance exercise (without balance and functional exercises), dance, or walking on the rate of falls.

The programs evaluated in the trials found to prevent falls included a total program duration that ranged from 5 weeks to 2.5 years, with total hours of exercise ranged from 6 to 312 h. Further program characteristics for types of exercise found to be effective in falls prevention have been carefully evaluated in a previous review [9]. There was a suggestion of a dose-response relationship of exercise on rate of falls (Fig. 2) but, unlike type of exercise, this did not reach statistical significance. The estimated 42% reduction in the rate of falls from programs that include a higher dose of exercise (when dichotomised at 3 h per week) and include exercises that target balance or function suggests that attention to both type and dose of exercise is important.

Despite our thorough search strategy, we acknowledge the possibility that some relevant trials, especially if they were published in languages other than English, may have been missed. Two review authors independently classified and assigned the exercise intervention categories to primary or secondary status using the ProFaNE guidelines [16]. We recognise there is some subjectivity in this classification system, particularly for those interventions containing more than one category of exercise. In the 2019 Cochrane Review [11], sensitivity analyses that tested the effects of re-categorising primary balance and functional exercise trials with a secondary component of strength training indicated that this did not substantively affect the results.

This update adds to the existing body of evidence by including nine additional studies to the 2019 Cochrane Review [11] and supports the findings of Gillespie 2012 [6] and Sherrington 2019 [11], whereby multiple component group-based exercise was found to reduce the rate of falls. In the recent work, we extended the findings of Gillespie 2012 [6] by recoding intervention programs and attempted to identify a primary exercise component for each included study. We only classified the intervention program in the study as ‘multiple component’ category if the intervention had an equal focus on each of the multiple components. As a result, fewer studies in this review were identified as multiple component programs and more studies were identified as balance and functional exercises. The present review also adds to our previous non-Cochrane review [8], that used a different methodology (multivariable meta-regression) yet reached similar conclusions about the importance of the inclusion of exercises that safely challenge balance in falls prevention exercise programs. The key difference in methods is that the Cochrane reviews undertake sub-group analyses for type of exercise by grouping programs according to their primary description. The meta-regression in our non-Cochrane review tests whether the inclusion of different features are associated with bigger effects. Other recent analyses have reached similar findings, including a large network meta-analysis [24].

Large studies are now needed to establish the impact of falls prevention interventions on fall-related fractures and falls requiring medical attention, as these are particularly costly to health systems and impactful for individuals. When developing priority topics for future research, the current evidence base should be considered in conjunction with the areas studied in the ongoing trials. Individual participant data meta-analysis could contribute further to the investigation of differential effects of exercise in people of different ages and baseline fall risks, as these are individual-level rather than trial-level characteristics. Further research is required to establish the effectiveness of falls prevention programs in emerging economies, where the burden of falls is increasing more rapidly than in high-income countries due to rapidly ageing populations [10]. There is an urgent need to investigate strategies to enhance implementation and scale-up of effective exercise-based falls prevention interventions into routine care of older people by healthcare professionals and community organisations. Systematic reviews of the characteristics of effective exercise interventions can guide clinicians and program providers in developing optimal interventions [9]. To enhance consistency of falls outcomes measurement in trials, studies should continue to use the consensus definition of a fall developed for trials in community-dwelling populations by ProFaNE [25] “an unexpected event in which the participant comes to rest on the ground, floor, or lower level”, consistent methods of falls ascertainment, and consistent measurement of adverse events in both groups throughout the trial period. There is a need to develop objective instruments for falls detection to replace the current reliance on self-reporting for falls. Future research should use the ProFaNE descriptors to categorise interventions [16], but should be clear how this was operationalised.

Current evidence about falls prevention suggests a targeted approach to exercise rather than more general promotion of physical activity. The importance of exercise in falls prevention suggests that greater attention be given to the widespread implementation of a life course approach to healthy ageing, i.e. lifelong exercise to maximise physical functioning in older age, as suggested by the World Health Organization [26]. Although trial follow-up ranged from 3 to 18 months in the main comparison, introducing falls prevention exercise habits in people in the general community are likely to have longer-term benefits. Notably, the duration of most of the exercise programs was 12 weeks or over and nearly one-third lasted a year or more. These findings also highlight the importance of ongoing exercise. As it is possible that interventions designed to increase physical activity could increase falls due to increased exposure to risk, we suggest that those undertaking trials of physical activity interventions in older people consider monitoring falls.

## Conclusion

In conclusion, this review confirms previous findings that exercise prevent falls in older adults. This updated review provides high-certainty evidence that well-designed exercise programs reduce the rate of falls among older people living in the community by approximately 25%. Greater provision and scale-up of these programs is an urgent challenge for the global health and exercise providers as well as social support systems.

## Supplementary Information

**Additional file 1.** Search strategy

**Additional file 2.** Categories of exercise (ProFaNE): definitions and applications

**Additional file 3: Table S1.** Characteristics and risk of bias assessment of the 116 included trials

**Additional file 4: Table S2.** Summary of findings: Rate of falls outcome (falls per person-years) for types of exercise

**Additional file 5: Table S3.** Components of studies in categories of exercise found to prevent falls

**Additional file 6: Table S4.** Components of studies in categories of exercise not found to prevent falls

## Data Availability

All the data generated or analysed in this study are included in this article and its supplementary information files, or in the published article included in the review as stated.

## Abbreviations

CI: Confidence interval
GRADE: Grading of Recommendations Assessment, Development and Evaluation
IQR: Interquartile range
ProFaNE: Prevention of Falls Network Europe
RaR: Pooled rate ratio
RCTs: Randomised controlled trials
