# Supplementary material for: Evidence on physical activity and falls prevention for people aged 65+ years: systematic review to inform the WHO guidelines on physical activity and sedentary behaviour
Source: Int J Behav Nutr Phys Act. 2020 Nov 26;17:144. doi: 10.1186/s12966-020-01041-3 (PMC7689963; doi:10.1186/s12966-020-01041-3)
Supplement: Supplementary file 1 — Additional file 1. Search strategy [file 12966_2020_1041_MOESM1_ESM.docx]

Additional file 1. Search strategy

***CENTRAL (CRS Online)***

#1 MESH DESCRIPTOR Accidental Falls EXPLODE ALL TREES
#2 (falls or faller*):TI,AB,KY
#3 #1 or #2
#4 MESH DESCRIPTOR Aged EXPLODE ALL TREES
#5 (senior* or elder* or old* or aged or ag?ing or postmenopausal or community dwelling):TI,AB,KY
#6 #4 or #5
#7 #3 and #6

***MEDLINE (Ovid Interface)***

1 Accidental Falls/
2 (falls or faller*1).tw.
3 or/1-2
4 exp Aged/
5 (senior*1 or elder* or old* or aged or ag?ing or postmenopausal or community dwelling).tw.
6 or/4-5
7 3 and 6
8 Randomized controlled trial.pt.
9 Controlled clinical trial.pt.
10 randomized.ab.
11 placebo.ab.
12 Clinical trials as topic/
13 randomly.ab.
14 trial.ti.
15 8 or 9 or 10 or 11 or 12 or 13 or 14
16 exp Animals/ not Humans/
17 15 not 16
18 7 and 17

***Embase (Ovid Interface)***

1 Falling/
2 (falls or fallers).tw.
3 or/1-2
4 exp Aged/
5 (senior*1 or elder* or old* or aged or ag?ing or postmenopausal or community dwelling).tw.
6 or/4-5
7 3 and 6
8 exp Randomized Controlled Trial/ or exp Single Blind Procedure/ or exp Double Blind Procedure/ or Crossover Procedure/
9 (random* or RCT or placebo or allocat* or crossover* or 'cross over' or trial or (doubl* adj1 blind*) or (singl* adj1 blind*)).ti,ab.
10 8 or 9
11 (exp Animal/ or animal.hw. or Nonhuman/) not (exp Human/ or Human cell/ or (human or humans).ti.)
12 10 not 11
13 7 and 12

***CINAHL (Ebsco)***

S1 (MH "Accidental Falls")
S2 TI ( falls or faller* ) OR AB ( falls or faller* )
S3 S1 OR S2
S4 (MH "Aged+")
S5 TI ( senior* or elder* or old* or aged or ag?ing or postmenopausal or community dwelling ) OR AB ( senior* or elder* or old* or aged or ag?ing or postmenopausal or community dwelling )
S6 S4 OR S5
S7 S3 AND S6
S8 PT Clinical Trial
S9 (MH "Clinical Trials+")
S10 TI clinical trial* OR AB clinical trial*
S11 TI ( (single blind* or double blind*) ) OR AB ( (single blind* or double blind*) )
S12 TI random* OR AB random*
S13 S8 OR S9 OR S10 OR S11 OR S12
S14 S7 AND S13

***PEDro***

Advanced search option selected

Abstract and Title: fall*
Method: clinical trial
Sub discipline: gerontology

New record added since: (date of last review entered here)

***ClinicalTrials.gov***

(prevent OR reduce OR reduction OR risk) AND (fall OR fallers) AND (exercise OR training)

***WHO ICTRP***

prevent* AND fall* AND exercise* OR reduc* AND fall* AND exercise* OR risk* AND fall* AND exercise* OR prevent* AND fall* AND train* OR reduc* AND fall* AND train* OR risk* AND fall* AND exercise*
