# Supplementary material for: Evidence on physical activity and falls prevention for people aged 65+ years: systematic review to inform the WHO guidelines on physical activity and sedentary behaviour
Source: Int J Behav Nutr Phys Act. 2020 Nov 26;17:144. doi: 10.1186/s12966-020-01041-3 (PMC7689963; doi:10.1186/s12966-020-01041-3)
Supplement: Supplementary file 2 — Additional file 2. Categories of exercise (ProFaNE): definitions and applications [file 12966_2020_1041_MOESM2_ESM.docx]

Additional file 2. Categories of exercise (ProFaNE): definitions and applications

| **Exercise category** | **ProFaNE description**(1) | **How the category criteria were applied in this review^a^** |
| --- | --- | --- |
| Gait, balance, and functional training | Gait training involves specific correction of walking technique (e.g. posture, stride length and cadence) and changes of pace, level and direction. Balance training involves the efficient transfer of bodyweight from one part of the body to another or challenges specific aspects of the balance systems (e.g. vestibular systems). Balance retraining activities range from the re-education of basic functional movement patterns to a wide variety of dynamic activities that target more sophisticated aspects of balance. Functional training uses functional activities as the training stimulus, and is based on the theoretical concept of task specificity. All gait, balance and functional training should be based on an assessment of the participant’s abilities prior to starting the program; tailoring of the intervention to the individuals abilities; and progression of the exercise program as ability improves  Illustrative examples:   - Gait training - Heel raises, toe raises, walking on the toes/ heels, heel to toe walking, walking backward, forwards, sideways, turning, bending, stepping (stair climbing where available) side stepping. - Vestibular and proprioceptive retraining exercises (e.g. Cawthorne Cooksey exercises, different head and eye positions etc) - Specific exercises involving changes in pace, level, head and eye gaze, obstacle courses etc. - Ball exercises (and other co-ordination and reaction activities) - Foot eye coordination - Psychomotor performance (reaction time) - Obstacle courses - Standing on unstable surface - Walking in line - Reactive games - Dynamic balance exercises include knee bends (squats), calf raises and toe raises - Static balance exercises include standing in one leg or tandem standing - Walking exercises include turning, tandem, backward and sideways walking | Selected as exercise category if the intervention met the baseline assessment, tailoring and progression criteria. Selected as primary category for interventions where most exercises were conducted standing and where the intervention focus and most time spent was on exercise in this category |
| Strength/resistance (including power) | The term 'resistance training' covers all types of weight training i.e. contracting the muscles against a resistance to ‘overload’ and bring about a training effect in the muscular system. The resistance is an external force, which can be one’s own body placed in an unusual relationship to gravity (e.g. prone back extension) or an external resistance (e.g. free weight). All strength/resistance training should be based on an assessment of the participant’s abilities prior to starting the program; tailoring the intervention to the individual's abilities; and progression of the exercise program as ability improves.  Illustrative examples:   - weight training (free weights, fixed resistance equipment, resistance/ theraband bands and also body weight in the early stages of training) - Functional training with added weight (ie. weighted shopping bags etc) - Propulsion (explosive movement) training for power (eg. jumping) - Pilates resistance exercises - Weight lifting with free weights - Exercise on machines - Cable pulleys | Selected as exercise category if the intervention met the baseline assessment, tailoring and progression criteria. Selected as primary category for interventions where additional resistance was used or where it was clear that overload was sufficient without external resistance and where the intervention focus and most time spent was on exercise in this category |
| Flexibility | Flexibility training is the planned process by which stretching exercises are practised and progressed to restore or maintain the optimal range of movement (ROM) available to a joint or joints. The ranges of motion used by flexibility programs may vary from restoration/maintenance of the entire physiological range of motion, or alternatively, maintenance of range that is essential to mobility or other functions  Illustrative examples:   - Static Stretches (eg. hamstring/calf/chest/side stretch) - Pilates flexibility training (eg static and moving stretches) - Yoga | Selected as exercise category if the intervention met the progression of stretching criterion. Selected as primary category for interventions where flexibility training was a stated aim of the intervention and where the intervention focus and most time spent was on exercise in this category |
| 3D | 3D training involves constant movement in a controlled, fluid, repetitive way through all three spatial planes or dimensions (forward and back, side to side, and up and down). Tai Chi and Qi Gong incorporate specific weight transferences and require upright posture and subtle changes of head position and gaze direction. Dance involves a wide range of dynamic movement qualities, speeds and patterns  Illustrative examples:   - Tai Chi - Qi Gong - Dance | Selected as exercise category if the intervention involved Tai Chi or dance. Selected as primary category for interventions where the intervention focus and most time spent was on exercise in this category |
| General physical activity | Physical activity is any bodily movement produced by skeletal muscle contraction resulting in a substantial increase in energy expenditure. Physical activity has both occupational, transportation and recreational components and includes pursuits like golf, tennis, and swimming. It also includes other active pastimes like gardening, cutting wood, and carpentry. Physical activity can provide progressive health benefits and is a catalyst for improving health attitudes, health habits, and lifestyle. Increasing habitual physical activity should be with specific recommendations as to duration, frequency and intensity if a physical or mental health improvement is indicated  Illustrative examples:   - Walking indoors and outdoors - Swimming and cycling. | Selected as exercise category if the intervention included unstructured physical activity. We classed programs that included unstructured walking as this category. Selected as primary category for interventions where the intervention focus and most time spent was on exercise in this category |
| Endurance | Endurance training is aimed at cardiovascular conditioning and is aerobic in nature and simultaneously increases the heart rate and the return of blood to the heart.  Illustrative examples:   - Walking (30 mins 5 x p/w at a moderate/brisk pace) - Cycle Ergometer - Treadmill walking - Rowing machines - Continuous marching etc. during exercise class - Brisk-walking - Jogging - Interval training/speed-play/Fartlek training | Selected as exercise category if the intervention focused on structured aerobic training. We classed programs that included treadmill walking as this category. Selected as primary category for interventions where the intervention focus and most time spent was on exercise in this category |
| Other | Other kinds of exercises not described | Selected as exercise category if the intervention did not meet the other categories listed and where the intervention focus and most time spent was on exercise in this category |

**Reference**

1. Lamb SE, Becker C, Gillespie LD, Smith JL, Finnegan S, Potter R, et al. Reporting of complex interventions in clinical trials: development of a taxonomy to classify and describe fall-prevention interventions. Trials. 2011;12:125.
