# Supplementary material for: Evidence on physical activity and falls prevention for people aged 65+ years: systematic review to inform the WHO guidelines on physical activity and sedentary behaviour
Source: Int J Behav Nutr Phys Act. 2020 Nov 26;17:144. doi: 10.1186/s12966-020-01041-3 (PMC7689963; doi:10.1186/s12966-020-01041-3)
Supplement: Supplementary file 3 — Additional file 3: Table S1. Characteristics and risk of bias assessment of the 116 included trials [file 12966_2020_1041_MOESM3_ESM.docx]

Supplementary Table 1: Characteristics and risk of bias assessment of the 116 included trials

| **First author,**  **year** | **Sample size** | **Trial location** | **Age** | **Gender (% women)** | **Falls risk at enrolment^a^** | **Inclusion criteria related to falls** | **Good adherence^b^** | **Risk of bias assessment^c^** | | | | | | |
| --- | --- | --- | --- | --- | --- | --- | --- | --- | --- | --- | --- | --- | --- | --- |
|  |  |  |  |  |  |  |  | **Sequence generation** | **Allocation concealment** | **Blinding of participants and personnel** | **Blinding of outcome assessment** | **Incomplete outcome data** | **Selective outcome data** | **Ascertainment bias** |
| Almeida, 2013 (1) | 119 | Brazil | 79 | 83^d^ | 1 | Previous falls | NR | 2 | 2 | 2 | 2 | 3 | 3 | 3 |
| Ansai, 2015 (2) | 69 | Brazil | 82 | 68 | 1 | All > 80 years | N | 1 | 1 | 2 | 3 | 1 | 3 | 2 |
| Arantes, 2015 (3) | 30 | Brazil | 73 | 100 | 1 | Previous falls | Y | 2 | 2 | 2 | 2 | 2 | 3 | 2 |
| †Arkkukangas, 2019 (21) | 175 | Sweden | 83 | 70 | 0 | - | NR | 1 | 1 | 2 | 1 | 1 | 1 | 1 |
| Ballard, 2004 (4) | 40 | USA | 73 | 100 | 1 | Previous falls | Y | 2 | 2 | 2 | 3 | 1 | 3 | 3 |
| †Barclay, 2018 (5) | 9 | Canada | 76 | 78 | 0 | - | Y | 1 | 2 | 2 | 1 | 1 | 1 | 1 |
| Barker, 2016 (6) | 53 | Australia | 69 | 88 | 1 | Other assessment | Y | 1 | 1 | 2 | 2 | 2 | 1 | 1 |
| Barnett, 2003 (7) | 163 | Australia | 75 | 67 | 1 | Poor balance or lower limb weakness or slow reaction time | Y | 2 | 1 | 2 | 2 | 1 | 2 | 2 |
| †Bernardelli, 2019 (8) | 186 | Italy | 76 | 80 | 0 | - | Y | 1 | 2 | 2 | 3 | 3 | 1 | 2 |
| Beyer, 2007 (9) | 65 | Denmark | 78 | 100 | 1 | All > 80 years or previous falls | Y | 1 | 2 | 2 | 2 | 2 | 3 | 1 |
| Boongird, 2017 (10) | 439 | Thailand | 74 | 83 | 1 | Poor balance | Y | 1 | 1 | 2 | 1 | 1 | 1 | 1 |
| Brown, 2002 (11) | 99 | Australia | 84^e^ | 79 | 0 | - | Y | 1 | 1 | 2 | 2 | 3 | 3 | 1 |
| Buchner, 1997 (12) | 105 | USA | 75 | 51 | 1 | Lower limb weakness or impaired gait | Y | 1 | 2 | 2 | 2 | 2 | 2 | 1 |
| Bunout, 2005 (13) | 298 | Chile | 75 | 71 | 0 | - | N | 1 | 2 | 2 | 2 | 2 | 3 | 2 |
| Campbell, 1997 (14) | 233 | New Zealand | 84 | 100 | 1 | All > 80 years | NR | 1 | 1 | 2 | 3 | 1 | 2 | 1 |
| Carter, 2002 (15) | 93 | Canada | 69 | 100 | 0 | - | Y | 1 | 2 | 2 | 1 | 1 | 3 | 1 |
| Cerny, 1998 (16) | 28 | USA | 71 | NR | 0 | - | NR | 1 | 3 | 2 | 2 | 1 | 3 | 3 |
| Clegg, 2014 (17) | 84 | UK | 79 | 71 | 1 | Recent rehabilitation | N | 1 | 1 | 2 | 2 | 2 | 1 | 2 |
| Clemson, 2010 (18) | 34 | Australia | 82 | 47 | 1 | Previous falls | NR | 1 | 1 | 2 | 2 | 1 | 2 | 1 |
| Clemson, 2012 (19) | 317 | Australia | 83 | 58 | 1 | Previous falls | Y | 1 | 1 | 2 | 1 | 2 | 1 | 1 |
| Cornillon, 2002 (20) | 303 | France | 71 | 83 | 0 | - | Y | 1 | 2 | 2 | 2 | 1 | 2 | 1 |
| Dadgari, 2016 (21) | 551 | Iran | 71 | 49 | 1 | Previous falls | NR | 2 | 2 | 2 | 2 | 3 | 2 | 3 |
| Dangour, 2011 (22) | 984 | Chile | 66 | 68 | 0 | - | N | 1 | 3 | 2 | 3 | 3 | 3 | 3 |
| Davis, 2011 (23) | 155 | Canada | 70 | 100 | 0 | - | NR | 1 | 1 | 1 | 1 | 1 | 3 | 1 |
| Day, 2002 (24) | 272 | Australia | 76 | 60 | 0 | - | Y | 1 | 1 | 2 | 1 | 1 | 2 | 1 |
| Day, 2015 (25) | 503 | Australia | 70 | 70 | 1 | Poor mobility | Y | 1 | 1 | 2 | 1 | 1 | 1 | 1 |
| Duque, 2013 (26) | 60 | Australia | 77 | 62 | 1 | Previous falls or poor balance | Y | 2 | 2 | 2 | 1 | 1 | 3 | 3 |
| Ebrahim, 1997 (27) | 165 | UK | 67 | 100 | 0 | - | Y | 1 | 2 | 3 | 3 | 3 | 2 | 2 |
| El-Khoury, 2015 (28) | 706 | France | 80 | 100 | 1 | Poor balance | N | 1 | 1 | 2 | 1 | 1 | 1 | 1 |
| Fiatarone, 1997 (29) | 34 | USA | 82 | 94 | 1 | Functional limitation | NR | 2 | 2 | 2 | 2 | 2 | 3 | 2 |
| Freiberger, 2007 (30) | 134 | Germany | 76 | 44 | 1 | Previous falls or fear of falling | Y | 1 | 2 | 2 | 2 | 1 | 3 | 1 |
| †Gallo, 2018 (31) | 69 | USA | 79 | 46 | 0 | - | NR | 2 | 2 | 2 | 2 | 3 | 1 | 2 |
| Gill, 2016 (32) | 1635 | USA | 79 | 67 | 1 | Functional limitation | Y | 1 | 1 | 2 | 2 | 2 | 3 | 3 |
| Grahn Kronhed, 2009 (33) | 65 | Sweden | 71 | 100 | 0 | - | Y | 1 | 1 | 2 | 1 | 1 | 3 | 1 |
| Gschwind, 2015 (34) | 153 | Australia, Spain, Germany | 75 | 61 | 0 | - | Y | 1 | 2 | 2 | 1 | 1 | 3 | 1 |
| Haines, 2009 (35) | 53 | Australia | 81 | 60 | 1 | Recent hospitalisation or use mobility aids | N | 1 | 1 | 2 | 1 | 1 | 1 | 1 |
| Halvarsson, 2013 (36) | 59 | Sweden | 77 | 71 | 1 | Previous falls or fear of falling | Y | 1 | 2 | 2 | 3 | 2 | 3 | 3 |
| Halvarsson, 2016 (37) | 96 | Sweden | 76 | 98 | 1 | Previous falls or fear of falling | Y | 1 | 2 | 3 | 3 | 2 | 3 | 3 |
| Hamrick, 2017 (38) | 43 | USA | 70 | 79 | 0 | - | Y | 2 | 2 | 2 | 1 | 1 | 2 | 3 |
| Hauer, 2001 (39) | 57 | Germany | 82 | 100 | 1 | Recent rehabilitation | Y | 2 | 2 | 2 | 1 | 1 | 2 | 1 |
| Helbostad, 2004 (40) | 77 | Norway | 81 | 81 | 1 | Previous falls or use mobility aids | Y | 2 | 1 | 1 | 1 | 1 | 2 | 1 |
| Hirase, 2015 (41) | 93 | Japan | 82 | 70 | 1 | Other assessment | Y | 2 | 2 | 2 | 3 | 1 | 3 | 1 |
| Huang, 2010 (42) | 115 | Taiwan | 72^d^ | 30^d^ | 0 | - | NR | 2 | 3 | 2 | 2 | 3 | 3 | 2 |
| Hwang, 2016 (43) | 456 | Taiwan | 72 | 67 | 1 | Previous falls | Y | 1 | 1 | 2 | 1 | 3 | 2 | 1 |
| Iliffe, 2015 (44) | 1254 | UK | 73 | 62 | 0 | - | N | 1 | 1 | 2 | 3 | 3 | 2 | 3 |
| Irez, 2011 (45) | 60 | Turkey | 75 | 100 | 0 | - | Y | 2 | 2 | 2 | 3 | 1 | 3 | 1 |
| Iwamoto, 2009 (46) | 68 | Japan | 76 | 90 | 0 | - | Y | 2 | 2 | 2 | 3 | 1 | 3 | 3 |
| Kamide, 2009 (47) | 57 | Japan | 71 | 100 | 0 | - | Y | 1 | 2 | 3 | 2 | 3 | 3 | 3 |
| Karinkanta, 2007 (48) | 149 | Finland | 72 | 100 | 0 | - | Y | 1 | 1 | 2 | 2 | 1 | 3 | 3 |
| Kemmler, 2010 (49) | 246 | Germany | 69 | 100 | 0 | - | Y | 1 | 1 | 1 | 1 | 1 | 3 | 1 |
| Kerse, 2010 (50) | 193 | New Zealand | 81 | 59 | 0 | - | Y | 1 | 2 | 3 | 1 | 1 | 2 | 3 |
| Kim, 2014 (51) | 105 | Japan | 78 | 100 | 1 | Previous falls | Y | 1 | 2 | 2 | 1 | 1 | 3 | 2 |
| Korpelainen, 2006 (52) | 160 | Finland | 73 | 100 | 0 | - | Y | 1 | 1 | 2 | 1 | 1 | 3 | 3 |
| Kovacs, 2013 (53) | 76 | Hungary | 68 | 100 | 0 | - | Y | 2 | 1 | 2 | 1 | 1 | 2 | 1 |
| Kwok, 2016 (54) | 80 | Singapore | 80 | 85 | 1 | Functional limitation | Y | 2 | 2 | 2 | 1 | 1 | 3 | 1 |
| Kyrdalen, 2014 (55) | 125 | Norway | 83 | 73 | 1 | Previous falls | Y | 1 | 1 | 3 | 3 | 3 | 3 | 3 |
| LaStayo 2017 (56) | 134 | USA | 76 | 65 | 1 | Previous falls | Y | 2 | 2 | 2 | 3 | 2 | 2 | 1 |
| Latham, 2003 (57) | 243 | Australia, New Zealand | 79 | 53 | 1 | Recent hospitalisation | Y | 1 | 1 | 1 | 1 | 2 | 2 | 2 |
| Lehtola, 2000 (58) | 131 | Finland | 72 | 80 | 0 | - | Y | 2 | 2 | 2 | 2 | 3 | 1 | 2 |
| Li, 2005 (59) | 256 | USA | 77 | 70 | 0 | - | Y | 1 | 2 | 2 | 1 | 3 | 2 | 1 |
| †Li, 2018 (60) | 670 | USA | 78 | 65 | 1 | Fall history/assessed risk of falls or reduced mobility | Y | 1 | 2 | 2 | 1 | 1 | 1 | 1 |
| Lin, 2007 (61) | 100 | Taiwan | 77 | 51 | 1 | Previous falls | NR | 2 | 2 | 2 | 2 | 3 | 3 | 1 |
| †Lipsitz, 2019 (62) | 180 | USA | 75 | 67 | 0 | - | Y | 1 | 2 | 2 | 1 | 3 | 1 | 1 |
| Liston, 2014 (63) | 21 | UK | 78 | 85 | 1 | Previous falls | NR | 1 | 2 | 2 | 2 | 3 | 3 | 3 |
| Liu-Ambrose, 2004 (64) | 104 | Canada | 79 | 100 | 0 | - | Y | 2 | 2 | 2 | 2 | 1 | 3 | 1 |
| Liu-Ambrose, 2008 (65) | 74 | Canada | 82 | 70 | 1 | Previous falls | Y | 1 | 1 | 2 | 3 | 3 | 2 | 1 |
| †Liu-Ambrose, 2019 (66) | 345 | Canada | 82 | 67 | 1 | Previous fall | Y | 1 | 1 | 2 | 2 | 1 | 1 | 1 |
| Logghe, 2009 (67) | 269 | Netherlands | 77 | 71 | 1 | Previous falls or poor balance or poor mobility or dizziness or diuretics use | Y | 1 | 1 | 2 | 1 | 1 | 2 | 1 |
| Lord, 1995 (68) | 197 | Australia | 72 | 100 | 0 | - | Y | 2 | 2 | 2 | 3 | 2 | 2 | 3 |
| Lord, 2003 (69) | 551 | Australia | 80 | 86 | 0 | - | N | 2 | 3 | 2 | 3 | 3 | 2 | 1 |
| Lurie, 2013 (70) | 64 | USA | 80 | 58 | 1 | Other assessment | NR | 1 | 2 | 2 | 3 | 2 | 3 | 3 |
| Luukinen, 2007 (71) | 486 | Finland | 88 | 79 | 1 | Previous falls | NR | 1 | 2 | 2 | 1 | 3 | 2 | 3 |
| †Ma, 2019 (72) | 33 | Hong Kong | 70 | 84 | 0 | - | NR | 2 | 1 | 2 | 1 | 3 | 1 | 3 |
| Madureira, 2007 (73) | 66 | Brazil | 74 | 100 | 0 | - | Y | 2 | 2 | 2 | 1 | 1 | 3 | 2 |
| McMurdo, 1997 (74) | 118 | UK | 65 | 100 | 0 | - | Y | 2 | 2 | 2 | 2 | 3 | 2 | 2 |
| Means, 2005 (75) | 338 | USA | 74 | 57 | 0 | - | Y | 1 | 2 | 2 | 1 | 3 | 2 | 1 |
| Merom, 2016 (76) | 530 | Australia | 78 | 85 | 0 | - | Y | 1 | 2 | 2 | 1 | 1 | 1 | 1 |
| Miko, 2016 (77) | 100 | Hungary | 79 | 100 | 0 | - | NR | 2 | 2 | 2 | 2 | 1 | 2 | 1 |
| Mirelman, 2016 (78) | 152 | Belgium, Israel, Italy, Netherlands, UK | 83 | 35 | 1 | Previous falls | Y | 1 | 1 | 2 | 1 | 1 | 3 | 1 |
| Morgan, 2004 (79) | 294 | USA | 81 | 71 | 1 | Prolong bed rest | Y | 2 | 2 | 2 | 2 | 3 | 3 | 1 |
| Morone, 2016 (80) | 38 | Italy | 69 | 100 | 1 | Poor balance | NR | 1 | 1 | 2 | 2 | 3 | 3 | 2 |
| Morrison, 2018 (81) | 65 | USA | 67 | 48 | 0 | - | NR | 1 | 2 | 2 | 2 | 3 | 3 | 3 |
| Ng, 2015 (82, 83) | 98 | Singapore | 70 | 61 | 1 | Frail | Y | 1 | 1 | 2 | 1 | 1 | 3 | 3 |
| Nitz, 2004 (84) | 73 | Australia | 76 | 92 | 1 | Previous falls | NR | 1 | 2 | 2 | 1 | 3 | 3 | 1 |
| Okubo, 2016 (85) | 105 | Japan | 70 | 63 | 0 | - | Y | 1 | 2 | 2 | 3 | 2 | 3 | 1 |
| †Oliveira, 2019 (86) | 131 | Australia | 72 | 71 | 0 | - | NR | 1 | 1 | 2 | 1 | 1 | 1 | 1 |
| Park 2008 (87) | 50 | Korea | 69 | 100 | 0 | - | NR | 1 | 2 | 2 | 2 | 1 | 3 | 3 |
| Reinsch, 1992 (88) | 230 | USA | 74 | 80 | 0 | - | Y | 2 | 3 | 2 | 2 | 1 | 3 | 1 |
| Resnick, 2002 (89) | 20 | USA | 88 | 100 | 0 | - | Y | 1 | 2 | 2 | 2 | 3 | 3 | 2 |
| Robertson, 2001 (90) | 240 | New Zealand | 81 | 68 | 0 | - | Y | 1 | 1 | 2 | 1 | 1 | 2 | 1 |
| Rubenstein, 2000 (91) | 59 | USA | 74 | 0 | 1 | Previous falls or lower limb weakness or poor balance | Y | 1 | 2 | 2 | 3 | 1 | 2 | 2 |
| Sakamoto, 2013 (92) | 1365 | Japan | 81 | 82 | 1 | Poor balance | NR | 1 | 2 | 2 | 2 | 3 | 2 | 1 |
| Sales 2017 (93) | 66 | Australia | 73 | 69 | 1 | Previous falls or fear of falling | Y | 1 | 2 | 2 | 3 | 3 | 1 | 1 |
| Sherrington, 2014 (94) | 340 | Australia | 81 | 74 | 1 | Recent hospitalisation | Y | 1 | 1 | 2 | 1 | 1 | 1 | 1 |
| Shigematsu, 2008 (95) | 68 | Japan | 69 | 63 | 0 | - | Y | 1 | 2 | 1 | 3 | 1 | 2 | 1 |
| Siegrist, 2016 (96) | 378 | Germany | 78 | 74 | 1 | Poor balance or fear of falling | Y | 1 | 2 | 2 | 1 | 1 | 1 | 1 |
| Skelton, 2005 (97) | 81 | UK | 73 | 100 | 1 | Previous falls | Y | 1 | 2 | 2 | 1 | 1 | 2 | 1 |
| Smulders, 2010 (98) | 96 | Netherlands | 71 | 94 | 1 | Previous falls | Y | 2 | 2 | 2 | 1 | 1 | 2 | 1 |
| Steadman, 2003 (99) | 199 | UK | 83 | 82 | 1 | Poor balance | Y | 1 | 2 | 2 | 1 | 3 | 3 | 3 |
| Suzuki, 2004 (100) | 52 | Japan | 78 | 100 | 0 | - | Y | 2 | 2 | 2 | 2 | 2 | 2 | 3 |
| Taylor, 2012 (101) | 684 | New Zealand | 75 | 73 | 1 | Previous falls | Y | 1 | 1 | 2 | 1 | 1 | 2 | 1 |
| Trombetti, 2011 (102) | 134 | Switzerland | 76 | 96 | 1 | Previous falls or poor balance | Y | 1 | 1 | 2 | 2 | 1 | 2 | 1 |
| Uusi-Rasi, 2015 (103) | 205 | Finland | 74 | 100 | 1 | Previous falls | Y | 1 | 2 | 2 | 2 | 2 | 2 | 1 |
| Verrusio, 2017 (104) | 150 | Italy | 65 | 53 | 1 | Poor balance | NR | 1 | 2 | 2 | 2 | 1 | 3 | 1 |
| Vogler, 2009 (105) | 180 | Australia | 80 | 83 | 1 | Recent hospitalisation | Y | 1 | 1 | 2 | 1 | 1 | 3 | 1 |
| Voukelatos, 2007 (106) | 702 | Australia | 69 | 84 | 0 | - | Y | 1 | 2 | 2 | 1 | 1 | 2 | 1 |
| Voukelatos, 2015 (107) | 386 | Australia | 73 | 74 | 0 | - | NR | 1 | 1 | 2 | 2 | 2 | 2 | 1 |
| Weerdesteyn, 2006 (108) | 58 | Netherlands | 74 | 77 | 1 | Previous falls | Y | 2 | 2 | 2 | 3 | 1 | 2 | 1 |
| Wolf, 1996 (109) | 200 | USA | 76 | 81 | 0 | - | Y | 1 | 2 | 2 | 2 | 1 | 3 | 1 |
| Wolf, 2003 (110) | 311 | USA | 81 | 94 | 1 | Previous falls | Y | 2 | 2 | 2 | 1 | 1 | 2 | 1 |
| Woo, 2007 (111) | 180 | China | 69 | 50 | 0 | - | Y | 1 | 2 | 2 | 3 | 3 | 3 | 3 |
| Wu, 2010 (112) | 64 | USA | 76 | 84 | 1 | Previous falls | Y | 2 | 2 | 2 | 2 | 1 | 3 | 3 |
| Yamada, 2010 (113) | 60 | Japan | NR | NR | 0 | - | Y | 2 | 1 | 2 | 2 | 2 | 2 | 1 |
| Yamada, 2012 (114) | 157 | Japan | 86 | 81 | 0 | - | Y | 2 | 1 | 3 | 2 | 1 | 2 | 1 |
| Yamada, 2013 (115) | 264 | Japan | 77 | 57 | 0 | - | Y | 2 | 2 | 2 | 2 | 2 | 1 | 1 |
| Yang, 2012 (116) | 165 | Australia | 81 | 44 | 1 | Poor balance | N | 1 | 2 | 2 | 1 | 3 | 3 | 3 |
| **Total low risk (%)** | | | | | | | | **79 (68%)** | **42 (36%)** | **5 (4%)** | **48 (41%)** | **62 (53%)** | **22 (19%)** | **68 (59%)** |
| **Total unclear risk (%)** | | | | | | | | **37 (32%)** | **69 (59%)** | **105 (91%)** | **44 (38%)** | **21 (18%)** | **42 (36%)** | **18 (16%)** |
| **Total high risk (%)** | | | | | | | | **0**  **(0%)** | **5**  **(4%)** | **6 (5%)** | **24 (21%)** | **33 (28%)** | **52 (45%)** | **30 (26%)** |

^a^ Presence of a particular risk factor for falls was used as inclusion criteria of the trial (0= No specific risk; 1= Previous falls, poor balance, recent hospitalisation, reduced lower strength, poor mobility, use mobility aids, frail, prolong bed rest, recent rehabilitation, functional limitation, all participants greater than age 80); ^b^ Attendance rate exceeded 50% and/or 75% or more of the participants attended 50% or more sessions;

^c^ Assessed using the Cochrane Risk of Bias tool (117) (1 = Low risk; 2 = unclear risk; 3 = high risk)

^d^ In people lost to follow-up; ^e^ Determined using numbers in each age group; † indicates studies that were found in the updated search, all other studies included in the 2019 Cochrane review (n=108) (118); N=No, Y=Yes, NR=Not reported

**References of the included studies**

1. Almeida TL, Alexander NB, Nyquist LV, Montagnini ML, Santos ACS, Rodrigues GHP, et al. Minimally supervised multi-modal exercise to reduce falls risk among economically and educationally disadvantaged older adults. Journal of aging and physical activity. 2013;21(3):241-59.

2. Ansai JH, Aurichio TR, Goncalves R, Rebelatto JR. Effects of two physical exercise protocols on physical performance related to falls in the oldest old: A randomized controlled trial. Geriatrics & gerontology international. 2015;16(4):492-9.

3. Arantes PMM, Dias JMD, Fonseca FF, Oliveira AMB, Oliveira MC, Pereira LSM, et al. Effect of a Program Based on Balance Exercises on Gait, Functional Mobility, Fear of Falling, and Falls in Prefrail Older Women: A Randomized Clinical Trial. Topics in Geriatric Rehabilitation. 2015;31(2):113-20.

4. Ballard JE, McFarland C, Wallace LS, Holiday DB, Roberson G. The effect of 15 weeks of exercise on balance, leg strength, and reduction in falls in 40 women aged 65 to 89 years. Journal of the American Medical Women's Association (1972). 2004;59(4):255-61.

5. Barclay R, Webber S, Ripat J, Grant T, Jones CA, Lix LM, et al. Safety and feasibility of an interactive workshop and facilitated outdoor walking group compared to a workshop alone in increasing outdoor walking activity among older adults: a pilot randomized controlled trial. Pilot Feasibility Stud. 2018;4:179.

6. Barker AL, Talevski J, Bohensky MA, Brand CA, Cameron PA, Morello RT. Feasibility of Pilates exercise to decrease falls risk: a pilot randomized controlled trial in community-dwelling older people. Clinical rehabilitation. 2016;30(10):984-96.

7. Barnett A, Smith B, Lord SR, Williams M, Baumand A. Community-based group exercise improves balance and reduces falls in at-risk older people: a randomised controlled trial. Age Ageing. 2003;32(4):407-14.

8. Bernardelli G, Roncaglione C, Damanti S, Mari D, Cesari M, Marcucci M. Adapted physical activity to promote active and healthy ageing: the PoliFIT pilot randomized waiting list-controlled trial. Aging Clin Exp Res. 2019;31(4):511-8.

9. Beyer N, Simonsen L, Bülow J, Lorenzen T, Jensen DV, Larsen L, et al. Old women with a recent fall history show improved muscle strength and function sustained for six months after finishing training. Aging Clinical and Experimental Research. 2007;19(4):300-9.

10. Boongird C, Keesukphan P, Phiphadthakusolkul S, Rattanasiri S, Thakkinstian A. Effects of a simple home-based exercise program on fall prevention in older adults: A 12-month primary care setting, randomized controlled trial. Geriatrics & gerontology international. 2017;17(11):2157-63.

11. Brown A. Functional adaptation to exercise in elderly subjects. Perth: Curtin University of Technology; 2002.

12. Buchner DM, Cress ME, de Lateur BJ, Esselman PC, Margherita AJ, Price R, et al. The effect of strength and endurance training on gait, balance, fall risk, and health services use in community-living older adults. The journals of gerontology Series A, Biological sciences and medical sciences. 1997;52(4):M218-24.

13. Bunout D, Barrera G, Avendaño M, de la Maza P, Gattas V, Leiva L, et al. Results of a community-based weight-bearing resistance training programme for healthy Chilean elderly subjects. Age and Ageing. 2005;34(1):80-3.

14. Campbell AJ, Robertson MC, Gardner MM, Norton RN, Tilyard MW, Buchner DM. Randomised controlled trial of a general practice programme of home based exercise to prevent falls in elderly women. BMJ. 1997;315(7115):1065-9.

15. Carter ND, Khan KM, McKay HA, Petit MA, Waterman C, Heinonen A, et al. Community-based exercise program reduces risk factors for falls in 65- to 75-year-old women with osteoporosis: randomized controlled trial. Canadian Medical Association journal. 2002;167(9):997-1004.

16. Cerny K, Blanks R, Mohamed O, Schwab D, Robinson B, Russo A, et al. The effect of a multidimensional exercise program on strength, range of motion, balance and gait in the well elderly. Gait & Posture. 1998;7(2):185-6.

17. Clegg A, Barber S, Young J, Iliffe S, Forster A. The Home-based Older People's Exercise (HOPE) trial: a pilot randomised controlled trial of a home-based exercise intervention for older people with frailty. Age and Ageing. 2014;43(5):687-95.

18. Clemson L, Singh MF, Bundy A, Cumming RG, Weissel E, Munro J, et al. LiFE Pilot Study: A randomised trial of balance and strength training embedded in daily life activity to reduce falls in older adults. Australian Occupational Therapy Journal. 2010;57(1):42-50.

19. Clemson L, Fiatarone Singh MA, Bundy A, Cumming RG, Manollaras K, O’Loughlin P, et al. Integration of balance and strength training into daily life activity to reduce rate of falls in older people (the LiFE study): randomised parallel trial. BMJ. 2012;345:e4547.

20. Cornillon E, Blanchon MA, Ramboatsisetraina P, Braize C, Beauchet O, Dubost V, et al. Effectiveness of falls prevention strategies for elderly subjects who live in the community with performance assessment of physical activities (before-after). Annales de readaptation et de medecine physique : revue scientifique de la Societe francaise de reeducation fonctionnelle de readaptation et de medecine physique. 2002;45(9):493-504.

21. Dadgari A, Aizan Hamid T, Hakim MN, Chaman R, Mousavi SA, Poh Hin L, et al. Randomized control trials on Otago Exercise Program (OEP) to reduce falls among elderly community dwellers in Shahroud, Iran. Iranian Red Crescent Medical Journal. 2016;18(5):e26340.

22. Dangour AD, Albala C, Allen E, Grundy E, Walker DG, Aedo C, et al. Effect of a nutrition supplement and physical activity program on pneumonia and walking capacity in Chilean older people: a factorial cluster randomized trial. PLoS medicine. 2011;8(4):e1001023.

23. Davis JC, Robertson MC, Ashe MC, Liu-Ambrose T, Khan KM, Marra CA. Does a home-based strength and balance programme in people aged > or =80 years provide the best value for money to prevent falls? A systematic review of economic evaluations of falls prevention interventions. Br J Sports Med. 2010;44(2):80-9.

24. Day L, Fildes B, Gordon I, Fitzharris M, Flamer H, Lord S. Randomised factorial trial of falls prevention among older people living in their own homes. BMJ. 2002;325(7356):128.

25. Day L, Hill KD, Stathakis VZ, Flicker L, Segal L, Cicuttini F, et al. Impact of tai-chi on falls among preclinically disabled older people. A randomized controlled trial. Journal of the american medical directors association. 2015;16(5):420‐6.

26. Duque G, Boersma D, Loza-Diaz G, Hassan S, Suarez H, Geisinger D, et al. Effects of balance training using a virtual-reality system in older fallers. Clinical Interventions in Aging. 2013;8:257-63.

27. Ebrahim S, Thompson PW, Baskaran V, Evans K. Randomized placebo-controlled trial of brisk walking in the prevention of postmenopausal osteoporosis. Age Ageing. 1997;26(4):253-60.

28. El-Khoury F, Cassou B, Latouche A, Aegerter P, Charles M-A, Dargent-Molina P. Effectiveness of two year balance training programme on prevention of fall induced injuries in at risk women aged 75-85 living in community: Ossébo randomised controlled trial. BMJ : British Medical Journal. 2015;351:h3830.

29. Fiatarone M, O’Neill E, Doyle R, Clements K, editors. Efficacy of home-based resistance training in frail elders. Abstracts of the 16th Congress of the International Association of Gerontology; 1997; Bedford Park, South Australia: World Congress of Gerontology Inc.

30. Freiberger E, Menz HB, Abu-Omar K, Rutten A. Preventing falls in physically active community-dwelling older people: a comparison of two intervention techniques. Gerontology. 2007;53(5):298‐305.

31. Gallo E, Stelmach M, Frigeri F, Ahn D-H. Determining Whether a Dosage-Specific and Individualized Home Exercise Program With Consults Reduces Fall Risk and Falls in Community-Dwelling Older Adults With Difficulty Walking: A Randomized Control Trial. Journal of Geriatric Physical Therapy. 2018;41(3):161-72.

32. Gill TM, Pahor M, Guralnik JM, McDermott MM, King AC, Buford TW, et al. Effect of structured physical activity on prevention of serious fall injuries in adults aged 70-89: randomized clinical trial (LIFE Study). Bmj. 2016;352:i245.

33. Grahn Kronhed A-C, Hallberg I, Ödkvist L, Möller M. Effect of training on health-related quality of life, pain and falls in osteoporotic women. Advances in Physiotherapy. 2009;11(3):154-65.

34. Gschwind YJ, Eichberg S, Ejupi A, de Rosario H, Kroll M, Marston HR, et al. ICT-based system to predict and prevent falls (iStoppFalls): results from an international multicenter randomized controlled trial. European Review of Aging and Physical Activity. 2015;12:10.

35. Haines TP, Russell T, Brauer SG, Erwin S, Lane P, Urry S, et al. Effectiveness of a video-based exercise programme to reduce falls and improve health-related quality of life among older adults discharged from hospital: a pilot randomized controlled trial. Clinical rehabilitation. 2009;23(11):973-85.

36. Halvarsson A, Franzen E, Faren E, Olsson E, Oddsson L, Stahle A. Long-term effects of new progressive group balance training for elderly people with increased risk of falling - a randomized controlled trial. Clinical rehabilitation. 2013;27(5):450-8.

37. Halvarsson A, Oddsson L, Franzén E, Ståhle A. Long-term effects of a progressive and specific balance-training programme with multi-task exercises for older adults with osteoporosis: a randomized controlled study. Clinical rehabilitation. 2016;30(11):1049‐59.

38. Hamrick I, Mross P, Christopher N, Smith PD. Yoga's effect on falls in rural, older adults. Complementary therapies in medicine. 2017;35:57‐63.

39. Hauer K, Rost B, Rutschle K, Opitz H, Specht N, Bartsch P, et al. Exercise training for rehabilitation and secondary prevention of falls in geriatric patients with a history of injurious falls. J Am Geriatr Soc. 2001;49(1):10-20.

40. Helbostad JL, Sletvold O, Moe-Nilssen R. Effects of home exercises and group training on functional abilities in home-dwelling older persons with mobility and balance problems. A randomized study. Aging clinical and experimental research. 2004;16(2):113‐21.

41. Hirase T, Inokuchi S, Matsusaka N, Okita M. Effects of a balance training program using a foam rubber pad in community-based older adults: a randomized controlled trial. Journal of geriatric physical therapy (2001). 2015;38(2):62-70.

42. Huang H-C, Liu C-Y, Huang Y-T, Kernohan WG. Community-based interventions to reduce falls among older adults in Taiwan – long time follow-up randomised controlled study. Journal of Clinical Nursing. 2010;19(7‐8):959-68.

43. Hwang HF, Chen SJ, Lee‐Hsieh J, Chien DK, Chen CY, Lin MR. Effects of Home‐Based Tai Chi and Lower Extremity Training and Self‐Practice on Falls and Functional Outcomes in Older Fallers from the Emergency Department—A Randomized Controlled Trial. Journal of the American Geriatrics Society. 2016;64(3):518-25.

44. Iliffe S, Kendrick D, Morris R, Masud T, Gage H, Skelton D, et al. Multicentre cluster randomised trial comparing a community group exercise programme and home-based exercise with usual care for people aged 65 years and over in primary care. Health technology assessment (Winchester, England). 2014;18(49):vii-xxvii, 1-105.

45. Irez GB, Ozdemir RA, Evin R, Irez SG, Korkusuz F. Integrating pilates exercise into an exercise program for 65+ year-old women to reduce falls. Journal of sports science & medicine. 2011;10(1):105-11.

46. Iwamoto J, Suzuki H, Tanaka K, Kumakubo T, Hirabayashi H, Miyazaki Y, et al. Preventative effect of exercise against falls in the elderly: a randomized controlled trial. Osteoporosis international : a journal established as result of cooperation between the European Foundation for Osteoporosis and the National Osteoporosis Foundation of the USA. 2009;20(7):1233-40.

47. Kamide N, Shiba Y, Shibata H. Effects on balance, falls, and bone mineral density of a home-based exercise program without home visits in community-dwelling elderly women: a randomized controlled trial. Journal of physiological anthropology. 2009;28(3):115-22.

48. Karinkanta S, Heinonen A, Sievanen H, Uusi-Rasi K, Pasanen M, Ojala K, et al. A multi-component exercise regimen to prevent functional decline and bone fragility in home-dwelling elderly women: randomized, controlled trial. Osteoporosis international : a journal established as result of cooperation between the European Foundation for Osteoporosis and the National Osteoporosis Foundation of the USA. 2007;18(4):453-62.

49. Kemmler W, von Stengel S, Engelke K, Haberle L, Kalender WA. Exercise effects on bone mineral density, falls, coronary risk factors, and health care costs in older women: the randomized controlled senior fitness and prevention (SEFIP) study. Archives of Internal Medicine. 2010;170(2):179-85.

50. Kerse N, Hayman KJ, Moyes SA, Peri K, Robinson E, Dowell A, et al. Home-based activity program for older people with depressive symptoms: DeLLITE--a randomized controlled trial. Annals of family medicine. 2010;8(3):214-23.

51. Kim H, Yoshida H, Suzuki T. Falls and fractures in participants and excluded non-participants of a fall prevention exercise program for elderly women with a history of falls: 1-year follow-up study. Geriatrics & gerontology international. 2014;14(2):285-92.

52. Korpelainen R, Keinanen-Kiukaanniemi S, Heikkinen J, Vaananen K, Korpelainen J. Effect of exercise on extraskeletal risk factors for hip fractures in elderly women with low BMD: a population-based randomized controlled trial. Journal of bone and mineral research : the official journal of the American Society for Bone and Mineral Research. 2006;21(5):772-9.

53. Kovacs E, Prokai L, Meszaros L, Gondos T. Adapted physical activity is beneficial on balance, functional mobility, quality of life and fall risk in community-dwelling older women: a randomized single-blinded controlled trial. European journal of physical and rehabilitation medicine. 2013;49(3):301-10.

54. Kwok BC, Pua YH. Effects of WiiActive exercises on fear of falling and functional outcomes in community-dwelling older adults: a randomised control trial. Age and ageing. 2016;45(5):621‐7.

55. Kyrdalen IL, Moen K, Røysland AS, Helbostad JL. The Otago Exercise Program performed as group training versus home training in fall-prone older people: a randomized controlled Trial. Physiotherapy research international. 2014;19(2):108‐16.

56. LaStayo P, Marcus R, Dibble L, Wong B, Pepper G. Eccentric versus traditional resistance exercise for older adult fallers in the community: a randomized trial within a multi-component fall reduction program. BMC geriatrics. 2017;17(1):149.

57. Latham NK, Anderson CS, Lee A, Bennett DA, Moseley A, Cameron ID. A randomized, controlled trial of quadriceps resistance exercise and vitamin D in frail older people: the Frailty Interventions Trial in Elderly Subjects (FITNESS). Journal of the American Geriatrics Society. 2003;51(3):291-9.

58. Lehtola S, Hanninen L, Paatalo M. The incidence of falls during a six month exercise trial and four month follow-up among home dwelling persons aged 70–75 years. Liikunta Tiede. 2000;6:41-7.

59. Li F, Harmer P, Fisher KJ, McAuley E, Chaumeton N, Eckstrom E, et al. Tai Chi and Fall Reductions in Older Adults: A Randomized Controlled Trial. The Journals of Gerontology: Series A. 2005;60(2):187-94.

60. Li F, Harmer P, Fitzgerald K, Eckstrom E, Akers L, Chou L-S, et al. Effectiveness of a Therapeutic Tai Ji Quan Intervention vs a Multimodal Exercise Intervention to Prevent Falls Among Older Adults at High Risk of Falling. JAMA Internal Medicine. 2018;178(10):1301-10.

61. Lin MR, Wolf SL, Hwang HF, Gong SY, Chen CY. A randomized, controlled trial of fall prevention programs and quality of life in older fallers. Journal of the American Geriatrics Society. 2007;55(4):499-506.

62. Lipsitz LA, Macklin EA, Travison TG, Manor B, Gagnon P, Tsai T, et al. A Cluster Randomized Trial of Tai Chi vs Health Education in Subsidized Housing: The MI‐WiSH Study. Journal of the American Geriatrics Society. 2019;67(9):1812-9.

63. Liston MB, Alushi L, Bamiou DE, Martin FC, Hopper A, Pavlou M. Feasibility and effect of supplementing a modified OTAGO intervention with multisensory balance exercises in older people who fall: a pilot randomized controlled trial. Clinical rehabilitation. 2014;28(8):784‐93.

64. Liu-Ambrose T, Khan KM, Eng JJ, Janssen PA, Lord SR, McKay HA. Resistance and agility training reduce fall risk in women aged 75 to 85 with low bone mass: a 6-month randomized, controlled trial. Journal of the American Geriatrics Society. 2004;52(5):657-65.

65. Liu-Ambrose T, Donaldson MG, Ahamed Y, Graf P, Cook WL, Close J, et al. Otago home-based strength and balance retraining improves executive functioning in older fallers: a randomized controlled trial. Journal of the American Geriatrics Society. 2008;56(10):1821-30.

66. Liu-Ambrose T, Davis JC, Best JR, Dian L, Madden K, Cook W, et al. Effect of a Home-Based Exercise Program on Subsequent Falls Among Community-Dwelling High-Risk Older Adults After a Fall: A Randomized Clinical Trial. JAMA. 2019;321(21):2092-100.

67. Logghe IH, Zeeuwe PE, Verhagen AP, Wijnen-Sponselee RM, Willemsen SP, Bierma-Zeinstra SM, et al. Lack of effect of Tai Chi Chuan in preventing falls in elderly people living at home: a randomized clinical trial. Journal of the American Geriatrics Society. 2009;57(1):70-5.

68. Lord SR, Ward JA, Williams P, Strudwick M. The effect of a 12-month exercise trial on balance, strength, and falls in older women: a randomized controlled trial. Journal of the American Geriatrics Society. 1995;43(11):1198.

69. Lord SR, Castell S, Corcoran J, Dayhew J, Matters B, Shan A, et al. The effect of group exercise on physical functioning and falls in frail older people living in retirement villages: a randomized, controlled trial. Journal of the American Geriatrics Society. 2003;51(12):1685-92.

70. Lurie JD, Zagaria AB, Pidgeon DM, Forman JL, Spratt KF. Pilot comparative effectiveness study of surface perturbation treadmill training to prevent falls in older adults. BMC geriatrics. 2013;13:49.

71. Luukinen H, Lehtola S, Jokelainen J, Vaananen-Sainio R, Lotvonen S, Koistinen P. Pragmatic exercise-oriented prevention of falls among the elderly: a population-based, randomized, controlled trial. Preventive medicine. 2007;44(3):265-71.

72. Ma AWW, Wang HK, Chen DR, Chen YM, Chak YTC, Chan JWY, et al. Chinese Martial Art Training Failed to Improve Balance or Inhibit Falls in Older Adults. Percept Mot Skills. 2019;126(3):389-409.

73. Madureira MM, Takayama L, Gallinaro AL, Caparbo VF, Costa RA, Pereira RM. Balance training program is highly effective in improving functional status and reducing the risk of falls in elderly women with osteoporosis: a randomized controlled trial. Osteoporosis international : a journal established as result of cooperation between the European Foundation for Osteoporosis and the National Osteoporosis Foundation of the USA. 2007;18(4):419-25.

74. McMurdo MET, Mole PA, Paterson CR. Controlled trial of weight bearing exercise in older women in relation to bone density and falls. BMJ. 1997;314(7080):569.

75. Means KM, Rodell DE, O'Sullivan PS. Balance, mobility, and falls among community-dwelling elderly persons: effects of a rehabilitation exercise program. American journal of physical medicine & rehabilitation. 2005;84(4):238-50.

76. Merom D, Mathieu E, Cerin E, Morton RL, Simpson JM, Rissel C, et al. Social Dancing and Incidence of Falls in Older Adults: A Cluster Randomised Controlled Trial. PLoS medicine. 2016;13(8):e1002112.

77. Miko I, Szerb I, Szerb A, Poor G. Effectiveness of balance training programme in reducing the frequency of falling in established osteoporotic women: a randomized controlled trial. Clinical rehabilitation. 2017;31(2):217-24.

78. Mirelman A, Rochester L, Maidan I, Del Din S, Alcock L, Nieuwhof F, et al. Addition of a non-immersive virtual reality component to treadmill training to reduce fall risk in older adults (V-TIME): a randomised controlled trial. Lancet (London, England). 2016;388(10050):1170-82.

79. Morgan RO, Virnig BA, Duque M, Abdel-Moty E, Devito CA. Low-intensity exercise and reduction of the risk for falls among at-risk elders. The journals of gerontology Series A, Biological sciences and medical sciences. 2004;59(10):1062-7.

80. Morone G, Paolucci T, Luziatelli S, Iosa M, Piermattei C, Zangrando F, et al. Wii Fit is effective in women with bone loss condition associated with balance disorders: a randomized controlled trial. Aging clinical and experimental research. 2016;28(6):1187‐93.

81. Morrison S, Simmons R, Colberg SR, Parson HK, Vinik AI. Supervised balance training and Wii Fit-based exercises lower falls risk in older adults with type 2 diabetes. Journal of the american medical directors association. 2018;19(2):185.e7-13.

82. Ng TP, Feng L, Nyunt MS, Feng L, Niti M, Tan BY, et al. Nutritional, physical, cognitive, and combination interventions and frailty reversal among older adults: a randomized controlled trial. The American journal of medicine. 2015;128(11):1225-36.e1.

83. Arkkukangas M, Johnson ST, Hellstrom K, Soderlund A, Eriksson S, Johansson AC. A feasibility study of a randomised controlled trial comparing fall prevention using exercise with or without the support of motivational interviewing. Prev Med Rep. 2015;2:134-40.

84. Nitz JC, Choy NL. The efficacy of a specific balance-strategy training programme for preventing falls among older people: a pilot randomised controlled trial. Age and ageing. 2004;33(1):52‐8.

85. Okubo Y, Osuka Y, Jung S, Rafael F, Tsujimoto T, Aiba T, et al. Walking can be more effective than balance training in fall prevention among community-dwelling older adults. Geriatrics & gerontology international. 2016;16(1):118‐25.

86. Oliveira JS, Sherrington C, Paul SS, Ramsay E, Chamberlain K, Kirkham C, et al. A combined physical activity and fall prevention intervention improved mobility-related goal attainment but not physical activity in older adults: a randomised trial. Journal of Physiotherapy. 2019;65(1):16-22.

87. Park H, Kim KJ, Komatsu T, Park SK, Mutoh Y. Effect of combined exercise training on bone, body balance, and gait ability: a randomized controlled study in community-dwelling elderly women. Journal of bone and mineral metabolism. 2008;26(3):254‐9.

88. Reinsch S, MacRae P, Lachenbruch PA, Tobis JS. Attempts to prevent falls and injury: a prospective community study. Gerontologist. 1992;32(4):450-6.

89. Resnick B. Testing the effect of the WALC intervention on exercise adherence in older adults. Journal of gerontological nursing. 2002;28(6):40-9.

90. Robertson MC, Devlin N, Gardner MM, Campbell AJ. Effectiveness and economic evaluation of a nurse delivered home exercise programme to prevent falls. 1: Randomised controlled trial. BMJ. 2001;322(7288):697-701.

91. Rubenstein LZ, Josephson KR, Trueblood PR, Loy S, Harker JO, Pietruszka FM, et al. Effects of a group exercise program on strength, mobility, and falls among fall-prone elderly men. The Journals of Gerontology: Series A. 2000;55(6):M317-M21.

92. Sakamoto K, Endo N, Harada A, Sakada T, Tsushita K, Kita K, et al. Why not use your own body weight to prevent falls? A randomized, controlled trial of balance therapy to prevent falls and fractures for elderly people who can stand on one leg for ≤15s. Journal of Orthopaedic Science. 2013;18(1):110-20.

93. Sales M, Polman R, Hill KD, Levinger P. A novel exercise initiative for seniors to improve balance and physical Function. Journal of aging and health. 2017;29(8):1424‐43.

94. Sherrington C, Lord SR, Vogler CM, Close JC, Howard K, Dean CM, et al. A post-hospital home exercise program improved mobility but increased falls in older people: a randomised controlled trial. PLoS One. 2014;9(9):e104412.

95. Shigematsu R, Okura T, Nakagaichi M, Tanaka K, Sakai T, Kitazumi S, et al. Square-stepping exercise and fall risk factors in older adults: a single-blind, randomized controlled trial. Journals of gerontology Series A, Biological sciences and medical sciences. 2008;63(1):76‐82.

96. Siegrist M, Freiberger E, Geilhof B, Salb J, Hentschke C, Landendoerfer P, et al. Fall prevention in a primary care setting: The effects of a targeted complex exercise intervention in a cluster randomized trial. Deutsches Ärzteblatt International. 2016;113(21):365-72.

97. Skelton D, Dinan S, Campbell M, Rutherford O. Tailored group exercise (Falls Management Exercise — FaME) reduces falls in community-dwelling older frequent fallers (an RCT). Age and Ageing. 2005;34(6):636-9.

98. Smulders E, Weerdesteyn V, Groen BE, Duysens J, Eijsbouts A, Laan R, et al. Efficacy of a short multidisciplinary falls prevention program for elderly persons with osteoporosis and a fall history: a randomized controlled trial. Archives of physical medicine and rehabilitation. 2010;91(11):1705-11.

99. Steadman J, Donaldson N, Kalra L. A randomized controlled trial of an enhanced balance training program to improve mobility and reduce falls in elderly patients. Journal of the American Geriatrics Society. 2003;51(6):847‐52.

100. Suzuki T, Kim H, Yoshida H, Ishizaki T. Randomized controlled trial of exercise intervention for the prevention of falls in community-dwelling elderly Japanese women. J Bone Miner Metab. 2004;22(6):602-11.

101. Taylor D, Hale L, Schluter P, Waters DL, Binns EE, McCracken H, et al. Effectiveness of tai chi as a community-based falls prevention intervention: a randomized controlled trial. Journal of the american geriatrics society. 2012;60(5):841‐8.

102. Trombetti A, Hars M, Herrmann FR, Kressig RW, Ferrari S, Rizzoli R. Effect of music-based multitask training on gait, balance, and fall risk in elderly people: a randomized controlled trial. Arch Intern Med. 2011;171(6):525-33.

103. Uusi-Rasi K, Patil R, Karinkanta S, Kannus P, Tokola K, Lamberg-Allardt C, et al. Exercise and vitamin D in fall prevention among older women: a randomized clinical trial. JAMA internal medicine. 2015;175(5):703-11.

104. Verrusio W, Gianturco V, Cacciafesta M, Marigliano V, Troisi G, Ripani M. Fall prevention in the young old using an exoskeleton human body posturizer: a randomized controlled trial. Aging Clinical and Experimental Research. 2017;29(2):207-14.

105. Vogler CM, Sherrington C, Ogle SJ, Lord SR. Reducing risk of falling in older people discharged from hospital: a randomized controlled trial comparing seated exercises, weight-bearing exercises, and social visits. Archives of physical medicine and rehabilitation. 2009;90(8):1317-24.

106. Voukelatos A, Cumming RG, Lord SR, Rissel C. A randomized, controlled trial of tai chi for the prevention of falls: the Central Sydney tai chi trial. Journal of the American Geriatrics Society. 2007;55(8):1185-91.

107. Voukelatos A, Merom D, Sherrington C, Rissel C, Cumming RG, Lord SR. The impact of a home-based walking programme on falls in older people: the Easy Steps randomised controlled trial. Age Ageing. 2015;44(3):377-83.

108. Weerdesteyn V, Rijken H, Geurts AC, Smits-Engelsman BC, Mulder T, Duysens J. A five-week exercise program can reduce falls and improve obstacle avoidance in the elderly. Gerontology. 2006;52(3):131-41.

109. Wolf SL, Barnhart HX, Kutner NG, McNeely E, Coogler C, Xu T. Reducing frailty and falls in older persons: an investigation of Tai Chi and computerized balance training. Atlanta FICSIT Group. Frailty and Injuries: Cooperative Studies of Intervention Techniques. Journal of the American Geriatrics Society. 1996;44(5):489-97.

110. Wolf SL, Sattin RW, Kutner M, O'Grady M, Greenspan AI, Gregor RJ. Intense tai chi exercise training and fall occurrences in older, transitionally frail adults: a randomized, controlled trial. Journal of the American Geriatrics Society. 2003;51(12):1693-701.

111. Woo J, Hong A, Lau E, Lynn H. A randomised controlled trial of Tai Chi and resistance exercise on bone health, muscle strength and balance in community-living elderly people. Age Ageing. 2007;36(3):262-8.

112. Wu G, Keyes L, Callas P, Ren X, Bookchin B. Comparison of telecommunication, community, and home-based Tai Chi exercise programs on compliance and effectiveness in elders at risk for falls. Archives of physical medicine and rehabilitation. 2010;91(6):849‐56.

113. Yamada M, Tanaka B, Nagai K, Aoyama T, Ichihashi N. Trail-walking exercise and fall risk factors in community-dwelling older adults: preliminary results of a randomized controlled trial. Journal of the american geriatrics society. 2010;58(10):1946‐51.

114. Yamada M, Aoyama T, Arai H, Nagai K, Tanaka B, Uemura K, et al. Complex obstacle negotiation exercise can prevent falls in community-dwelling elderly Japanese aged 75 years and older. Geriatrics & gerontology international. 2012;12(3):461‐7.

115. Yamada M, Higuchi T, Nishiguchi S, Yoshimura K, Kajiwara Y, Aoyama T. Multitarget stepping program in combination with a standardized multicomponent exercise program can prevent falls in community-dwelling older adults: a randomized, controlled trial. Journal of the american geriatrics society. 2013;61(10):1669‐75.

116. Yang XJ, Hill K, Moore K, Williams S, Dowson L, Borschmann K, et al. Effectiveness of a targeted exercise intervention in reversing older people's mild balance dysfunction: a randomized controlled trial. Physical therapy. 2012;92(1):24-37.

**Other references:**

117. Higgins JPT, Green S, Cochrane Collaboration. Cochrane handbook for systematic reviews of interventions. Chichester, England ; Hoboken, NJ: Wiley-Blackwell; 2011. xxi, 649 p. p.

118. Sherrington C, Fairhall NJ, Wallbank GK, Tiedemann A, Michaleff ZA, Howard K, et al. Exercise for preventing falls in older people living in the community. Cochrane Database Syst Rev. 2019;1:CD012424.
