# Supplementary material for: Evidence on physical activity and falls prevention for people aged 65+ years: systematic review to inform the WHO guidelines on physical activity and sedentary behaviour
Source: Int J Behav Nutr Phys Act. 2020 Nov 26;17:144. doi: 10.1186/s12966-020-01041-3 (PMC7689963; doi:10.1186/s12966-020-01041-3)
Supplement: Supplementary file 4 — Additional file 4: Table S2. Summary of findings: Rate of falls outcome (falls per person-years) for types of exercise [file 12966_2020_1041_MOESM4_ESM.docx]

Supplementary Table 2. Summary of findings: Rate of falls outcome (falls per person-years) for types of exercise

| Type of exercise | Follow-up range | Illustrative comparative risks* (95% CI) | | Relative effect  (95% CI) | No. of participants (studies) | Certainty of the evidence (GRADE) | Comments |
| --- | --- | --- | --- | --- | --- | --- | --- |
|  |  | Assumed risk | Corresponding risk |  |  |  |  |
| Exercise^a^ (all types) versus control^b^ (e.g. usual activities) | 3 to 30 months | Control | Exercise (all types) | Rate ratio 0.77  (0.71 to 0.83)^d^ | 14.306  (64 RCTs) | High^e^ | Overall, there is a reduction of 23% (95%CI 17% to 29%) in the number of falls  Guide to the data:  If 1000 people were followed over 1 year, the number of falls in the overall population would be 655 (95% CI 604 to 706) compared with 850 in the group receiving usual care or attention control. In the unselected population, the corresponding data are 466 (95%CI 430 to 503) compared with 605 in the group receiving usual care or attention control. In the selected higher-risk population, the corresponding data are 993 (95%CI 915 to 1071) compared with 1290 in the control group |
|  |  | All studies population | |  |  |  |  |
|  |  | 850 per 1000^c^ | 655 per 1000  (604 to 706) |  |  |  |  |
|  |  | Not selected for high risk population | |  |  |  |  |
|  |  | 605 per 1000^c^ | 466 per 1000  (430 to 503) |  |  |  |  |
|  |  | Selected for high risk population | |  |  |  |  |
|  |  | 1290 per 1000 ^c^ | 993 per 1000  (915 to 1071) |  |  |  |  |
| Balance, and functional exercises^f^ versus control^b^ (e.g. usual activities) | 3 to 30 months | Control | Exercise (gait, balance, and functional training) | Rate ratio 0.76 (0.70 to 0.82) | 7989  (39 RCTs) | High^h^ | Overall, there is a reduction of 24% (95%CI 18% to 30%) in the number of falls  Guide to the data based on the all-studies estimate.  If 1000 people were followed over 1 year, the number of falls would be 646 (95% CI 595 to 689) compared with 850 in the group receiving usual care or attention control |
|  |  | All studies population | |  |  |  |  |
|  |  | 850 per 1000 ^g^ | 646 per 1000  (595 to 689) |  |  |  |  |
|  |  | Specific exercise population | |  |  |  |  |
|  |  | 865 per 1000 ^g^ | 657 per 1000  (606 to 709) |  |  |  |  |
| Resistance exercises^i^ versus control^b^ (e.g. usual activities) | 4 to 12 months | Control | Exercise (resistance training) | Rate ratio 1.14 (0.67 to 1.97) | 327  (5 RCTs) | Very low^k^ | The evidence is of very low certainty, hence we are uncertain of the findings of an increase of 14% (95% CI 33% reduction to 97% increase) in the number of falls  Guide to the data based on the all-studies estimate.  If 1000 people were followed over 1 year, the number of falls would be 969 (95% CI 570 to 1675) compared with 850 in the group receiving usual care or attention control |
|  |  | All studies population | |  |  |  |  |
|  |  | 850 per 1000 ^j^ | 969 per 1000  (570 to 1675) |  |  |  |  |
|  |  | Specific exercise population | |  |  |  |  |
|  |  | 630 per 1000 ^j^ | 719 per 1000  (423 to 1242) |  |  |  |  |
| 3D (Tai Chi) exercise^l^ versus control^b^ (e.g. usual activities) | 6 to 17 months | Control | Exercise (3D (Tai Chi)) | Rate ratio 0.77 (0.61 to 0.97) | 3169  (9 RCTs) | Moderate^n^ | Overall, there is probably be a reduction of 23% (95% CI 3% to 39%) in the number of falls  Guide to the data based on the all-studies estimate. If 1000 people were followed over 1 year, the number of falls is probably 655 (95% CI 519 to 825) compared with 850 in the group receiving usual care or attention control |
|  |  | All studies population | |  |  |  |  |
|  |  | 850 per 1000 ^m^ | 655 per 1000  (519 to 825) |  |  |  |  |
|  |  | Specific exercise population | |  |  |  |  |
|  |  | 1290 per 1000 ^m^ | 993 per 1000  (787 to 1251) |  |  |  |  |
| 3D (dance) exercise^o^ versus control^b^ (e.g. usual activities) | 12 months | Control | Exercise (3D [dance]) | Rate ratio 1.34 (0.98 to 1.83) | 522  (1 RCT) | Very low^q^ | The evidence is of very low certainty, hence we are uncertain of the findings of an increase of 34% (95% CI 2% reduction to 83% increase) in the number of falls  Guide to the data based on the all-studies estimate  If 1000 people were followed over 1 year, the number of falls may be 1139 (95% CI 833 to 1556) compared with 850 in the group receiving usual care or attention control |
|  |  | All studies population | |  |  |  |  |
|  |  | 850 per 1000 ^p^ | 1139 per 1000  (833 to 1556) |  |  |  |  |
|  |  | Specific exercise population | |  |  |  |  |
|  |  | 800 per 1000 ^p^ | 1072 per 1000  (784 to 1464) |  |  |  |  |
| General physical activity (including walking) training^r^ versus control^b^ (e.g. usual activities) | 12 to 24 months | Control | Exercise (general physical activity [including walking]) | Rate ratio 1.14 (0.66 to 1.97) | 441  (2 RCTs) | Very low^t^ | The evidence is of very low certainty, hence we are uncertain of the findings of an increase of 14% (95% CI 34% reduction to 97% increase) in the number of falls  Guide to the data based on the all-studies estimate  If 1000 people were followed over 1 year, the number of falls may be 969 (95% CI 561 to 1675) compared with 850 in the group receiving usual care or attention control |
|  |  | All studies population | |  |  |  |  |
|  |  | 850 per 1000 ^s^ | 969 per 1000  (561 to 1675) |  |  |  |  |
|  |  | Specific exercise population | |  |  |  |  |
|  |  | 670 per 1000 ^s^ | 764 per 1000  (443 to 1320) |  |  |  |  |
| Multiple categories of exercise (often including, as primary interventions: gait, balance, and functional (task) training plus resistance training^u^ versus control^b^ (e.g. usual activities) | 2 to 25 months | Control | Exercise (multiple types (including, as primary interventions: gait, balance, and functional (task) training plus resistance training)) | Rate ratio 0.72 (0.56 to 0.93)^r^ | 2283  (15 RCTs) | Moderate^w^ | Overall, there is probably a reduction of 28% (95% CI 7% to 44%) in the number of falls  Guide to the data based on the all-studies estimate  If 1000 people were followed over 1 year, the number of falls would probably be 612 (95%CI 476 to 791) compared with 850 in the group receiving usual care or attention control |
|  |  | All studies population | |  |  |  |  |
|  |  | 850 per 1000^v^ | 612 per 1000  (476 to 791) |  |  |  |  |
|  |  | Specific exercise population | |  |  |  |  |
|  |  | 1205 per 1000^v^ | 868 per 1000  (675 to 791) |  |  |  |  |

**CI**: confidence interval

**GRADE Working Group grades of evidence**

**High certainty:** We are very confident that the true effect lies close to that of the estimate of the effect

**Moderate certainty:** We are moderately confident in the effect estimate: The true effect is likely to be close to the estimate of the effect, but there is a possibility that it is substantially different

**Low certainty:** Our confidence in the effect estimate is limited: The true effect may be substantially different from the estimate of the effect

**Very low certainty:** We have very little confidence in the effect estimate: The true effect is likely to be substantially different from the estimate of effect

^a^ Exercise is a physical activity that is planned, structured and repetitive and aims to improve or maintain physical fitness. There is a wide range of possible types of exercise, and exercise programs of ten include one or more types of exercise. We categorised exercise based on the Prevention of Falls Network Europe (ProFaNE) taxonomy that classifies exercise type as: i) gait, balance, and functional training; ii) strength/ resistance (including power); iii) flexibility; iv) three- dimensional (3D) exercise (e.g. Tai Chi, Qigong, dance); v) general physical activity; vi) endurance; and vii) other kind of exercises. The taxonomy allows for more than one type of exercise to be delivered within a program.

^b^ A control intervention is one that is not thought to reduce falls, such as general health education, social visits, very gentle exercise, or ’sham’ exercise not expected to impact on falls.

^c^ The all-studies population risk was based on the number of events and the number of participants in the control group for this outcome over the 64 RCTs. We calculated the risk in the control group using the median falls per person-year for the subgroups of trials for which a) an increased risk of falls was not an inclusion criterion (32 RCTs, 6434 participants), or b) increased risk of falls was an inclusion criterion (32 RCTs, 7872 participants).

^d^ Subgroup analysis found no difference based on whether risk of falls was an inclusion criterion or not (test for subgroup differences: Chi2 = 0.1, df = 1, P = 0.75, I^2^ = 0%).

^e^ There was no downgrading, including for risk of bias, as results were essentially unchanged with removal of the trials with a high risk of bias on one or more items.

^f^ Using Prevention of Falls Network Europe (ProFaNE) taxonomy, gait, balance, and functional training is: gait training = specific correction of walking technique, and changes of pace, level and direction; balance training = transferring bodyweight from one part of the body to another or challenging specific aspects of the balance systems; functional training = functional activities, based on the concept of task specificity. Training is assessment-based, tailored and progressed. Exercise programs included in this analysis contained a single primary exercise category (gait, balance, and functional training); these exercise programs may also include secondary categories of exercise.

^g^ The all-studies population risk was based on the number of events and the number of participants in the control group for this outcome over the 64 all-exercise types RCTs. The specific exercise population risk was based on the number of events and the number of participants in the control group for this outcome over the 39 RCTs.

^h^ We did not downgrade for risk of bias, as results were essentially unchanged with the removal of the trials with a high risk of bias in one or more items.

^i^ Using Prevention of Falls Network Europe (ProFaNE) taxonomy, resistance training is any type of weight training (contraction of muscles against resistance to induce a training effect in the muscular system). Resistance is applied by body weight or external resistance. Training is assessment-based, tailored and progressed. Exercise programs included in this analysis had resistance training as the single primary exercise category; these exercise programs may also include secondary categories of exercise.

^j^ The all-studies population risk was based on the number of events and the number of participants in the control group for this outcome over the 64 all-exercise types RCTs. The specific exercise population risk was based on the number of events and the number of participants in the control group for this outcome over the 5 RCTs.

^k^ Downgraded by three levels due to risk of inconsistency (there was substantial heterogeneity (I² = 67%)), imprecision (wide CI due to small sample size), and risk of bias (removing studies with high risk of bias in one or more items had a marked impact on results).

^l^ Using Prevention of Falls Network Europe (ProFaNE) taxonomy, 3D (Tai Chi) training uses upright posture, specific weight transferences and movements of the head and gaze, during constant movement in a fluid, repetitive, controlled manner through three spatial planes. Exercise programs included in this analysis had 3D (Tai Chi) training as the single primary exercise category; these exercise programs may also include secondary categories of exercise.

^m^ The all-studies population risk was based on the number of events and the number of participants in the control group for this outcome over the 64 all-exercise types RCTs. The specific exercise population risk was based on the number of events and the number of participants in the control group for this outcome over the nine RCTs.

^n^ Downgraded by one level due to inconsistency (there was substantial heterogeneity (I² = 83%). There was no downgrading for risk of bias, as results were essentially unchanged with removal of the trials with a high risk of bias on one or more items.

^o^ Using Prevention of Falls Network Europe (ProFaNE) taxonomy, 3D (dance) training uses dynamic movement qualities, patterns and speeds whilst engaged in constant movement in a fluid, repetitive, controlled manner through three spatial planes. Exercise programs included in this analysis had 3D (dance) training as the single primary exercise category; these exercise programs may also include secondary categories of exercise.

^p^ The all-studies population risk was based on the number of events and the number of participants in the control group for this outcome over the 64 all-exercise types RCTs. The specific exercise population risk was based on the number of events and the number of participants in the control group for this outcome in the sole RCT.

^q^ Graded very low due to serious imprecision (only one cluster-RCT, with a wide CI due to small sample size).

^r^ Using Prevention of Falls Network Europe (ProFaNE) taxonomy, physical activity is any movement of the body, produced by skeletal muscle, that causes energy expenditure to be substantially increased. Recommendations regarding intensity, frequency and duration are required in order to increase performance. Exercise programs included in this analysis had general physical activity (including walking) training as the single primary exercise category; these exercise programs may also include secondary categories of exercise.

^s^ The all-studies population risk was based on the number of events and the number of participants in the control group for this outcome over the 64 all-exercise types RCTs. The specific exercise population risk was based on the number of events and the number of participants in the control group for this outcome in the two RCTs.

^t^ Downgraded by three levels due to inconsistency (there was substantial heterogeneity (I² = 67%)), imprecision (wide CI), and risk of bias (removing studies with high risk of bias on one or more items had a marked impact on results).

^u^ Exercise programs included in this analysis had more than one primary exercise category. We categorised exercise based on the Prevention of Falls Network Europe (ProFaNE) taxonomy that classifies exercise type as: i) gait, balance, and functional (task) training; ii) strength/ resistance (including power); iii) flexibility; iv) three-dimensional (3D) exercise (e.g. Tai Chi, Qigong, dance); v) general physical activity; vi) endurance; and vii) other kind of exercises. The programs of ten included, as the primary intervention, gait, balance, and functional (task) training plus resistance training. The exercise programs may also include secondary categories of exercise.

^v^ The all-studies population risk was based on the number of events and the number of participants in the control group for this outcome over the 64 all-exercise types RCTs. The specific exercise population risk was based on the number of events and the number of participants in the control group for this outcome over the 15 RCTs.

^w^ Downgraded by one level due to inconsistency (there was substantial heterogeneity (I² = 71%)). We did not downgrade for risk of bias, as results were essentially unchanged with removal of the trials at a high risk of bias in one or more items
