# Supplementary material for: Evidence on physical activity and falls prevention for people aged 65+ years: systematic review to inform the WHO guidelines on physical activity and sedentary behaviour
Source: Int J Behav Nutr Phys Act. 2020 Nov 26;17:144. doi: 10.1186/s12966-020-01041-3 (PMC7689963; doi:10.1186/s12966-020-01041-3)
Supplement: Supplementary file 5 — Additional file 5: Table S3. Components of studies in categories of exercise found to prevent falls [file 12966_2020_1041_MOESM5_ESM.docx]

Supplementary Table 3: Components of studies in categories of exercise found to prevent falls (9)

| **First author, year and interventions** | **Type of exercise according to ProFaNE classification^16 a^** | | | | | | | **Duration of intervention (weeks)** | **Hours of intervention** | **Delivery mode^b^** | **Participants per instructor^c^** | **Tailored to the individual initially** | **Progressed based on individual assessment** | **Tailored in intensity or type** |
| --- | --- | --- | --- | --- | --- | --- | --- | --- | --- | --- | --- | --- | --- | --- |
|  | **Balance or functional training** | **Strength or resistance training** | **Flexibility training** | **3D exercise** | **General physical activity** | **Endurance exercise** | **Other exercise** |  |  |  |  |  |  |  |
| **Gait/Balance/functional training** |  |  |  |  |  |  |  |  |  |  |  |  |  |  |
| Almeida 2013 Fully supervised group‐based balance and strength training (24) | P | S | S | ‐ | ‐ | ‐ | ‐ | 16 | 40 | 1 | NR | N | NR | N |
| Almeida 2013 Minimally supervised group‐based balance and strength training (24) | P | S | S | ‐ | ‐ | ‐ | ‐ | 16 | 32 | 4 | NR | N | NR | N |
| Arantes 2015 Group‐based balance training (26) | P | ‐ | ‐ | ‐ | ‐ | ‐ | ‐ | 12 | 12 | 1 | NR | Y | Y | Y |
| Arkkukangas 2019 Individual Otago Exercise Program (20, 139) | P | S | ‐ | ‐ | S | ‐ | ‐ | 52 | 84 | 3 | None | Y | Y | Y |
| Barnett 2003 Group‐based balance, strength and aerobic training (30) | P | S | ‐ | ‐ | ‐ | S | ‐ | 52 | 65 | 4 | NR | N | N | N |
| Boongird 2017 Individual Otago Exercise Program (33) | P | S | ‐ | ‐ | S | ‐ | ‐ | 52 | 104 | 3 | None | Y | Y | Y |
| Campbell 1997 Individual Otago Exercise Program (37) | P | S | ‐ | ‐ | S | ‐ | ‐ | 52 | 160 | 3 | None | Y | Y | Y |
| Clegg 2014 Individual balance and strength training (40) | P | S | ‐ | ‐ | ‐ | ‐ | ‐ | 12 | 54 | 3 | None | Y | Y | Y |
| Clemson 2010 LiFE (Lifestyle approach to reducing Falls through Exercise) program‐ balance and strength training embedded in daily life activities (41) | P | S | ‐ | ‐ | ‐ | ‐ | ‐ | 26 | 60 | 3 | None | Y | Y | Y |
| Clemson 2012 LiFE (Lifestyle approach to reducing Falls through Exercise) program- balance and strength training embedded in daily life activities (42) | P | S | ‐ | ‐ | ‐ | ‐ | ‐ | 52 | 87 | 3 | None | Y | Y | Y |
| Cornillon 2002 Group‐based balance and gait training (43) | P | ‐ | ‐ | ‐ | ‐ | ‐ | ‐ | 52 | 10 | 1 | NR | N | N | N |
| Dadgari 2016 Individual Otago Exercise Program (44) | P | S | ‐ | ‐ | S | ‐ | ‐ | 24 | 72 | 3 | None | Y | Y | Y |
| Dangour 2011 Group‐based balance and strength (45) | P | S | ‐ | ‐ | S | ‐ | ‐ | 104 | 104 | 1 | NR | N | N | N |
| Day 2002 Group‐based balance and strength (47) | P | S | S | ‐ | ‐ | ‐ | ‐ | 18 | 30 | 1 | NR | N | N | N |
| Duque 2013 Virtual reality balance training (49) | P | ‐ | ‐ | ‐ | ‐ | ‐ | S‐ visual‐  vestibular  rehab | 6 | 6 | 2 | 1 | N | Y | Y |
| El‐Khoury 2015 Group‐based balance and strength plus home practice (51) | P | S | S | ‐ | ‐ | ‐ | ‐ | 104 | 104 | 4 | NR | Y | Y | Y |
| Gschwind 2015 Individual balance and strength training using exergames (57) | P | S | ‐ | ‐ | ‐ | ‐ | ‐ | 16 | 12 | 3 | None | Y | Y | Y |
| Halvarsson 2013 Group‐based progressive balance training (59) | P | ‐ | ‐ | ‐ | ‐ | ‐ | ‐ | 12 | 27 | 1 | 3-3.5 | Y | Y | Y |
| Halvarsson 2016 Group‐based progressive balance training (60) | P | ‐ | ‐ | ‐ | ‐ | ‐ | ‐ | 12 | 27 | 1 | 6-10 | Y | Y | Y |
| Hamrick 2017 Home exercise group (61) | P | ‐ | S | ‐ | ‐ | ‐ | ‐ | 8 | 41 | 4 | 11-13 | Y | Y | Y |
| Hirase 2015 Group‐based balance training on foam rubber (64) | P | ‐ | ‐ | ‐ | ‐ | ‐ | ‐ | 16 | 16 | 4 | 10 | N | N | N |
| Hirase 2015 Group‐based balance training on stable surface (64) | P | ‐ | ‐ | ‐ | ‐ | ‐ | ‐ | 16 | 16 | 4 | 10 | N | N | N |
| Iliffe 2015 Group‐based FaME plus home training based on Otago Exercise Program (67) | P | S | ‐ | ‐ | S | ‐ | ‐ | 24 | 72 | 4 | 9-10 | Y | Y | Y |
| Iliffe 2015 Individual Otago Exercise Program (67) | P | S | ‐ | ‐ | S | ‐ | ‐ | 24 | 60 | 3 | None | Y | Y | Y |
| Iwamoto 2009 Group‐based balance and gait training (69) | P | ‐ | ‐ | ‐ | ‐ | ‐ | ‐ | 20 | 30 | 4 | NR | N | N | N |
| Karinkanta 2007 Group‐based balance and agility training (71) | P | ‐ | ‐ | ‐ | ‐ | ‐ | ‐ | 52 | 104 | 1 | NR | N | N | N |
| Kerse 2010 Individual Otago Exercise Program (73) | P | S | ‐ | ‐ | S | ‐ | ‐ | 26 | 67 | 3 | None | Y | Y | Y |
| Korpelainen 2006 Group‐based balance and strength training plus home practice (75) | P | S | ‐ | ‐ | ‐ | ‐ | ‐ | 130 | 173 | 4 | 5-8 | Y | N | Y |
| Kovacs 2013 Group‐based balance and strength based on Otago Exercise Program (76) | P | S | ‐ | ‐ | S | ‐ | ‐ | 25 | 52 | 1 | 30-38 | Y | Y | Y |
| Lin 2007 Individual balance, strength and flexibility training (84) | P | S | S | ‐ | ‐ | ‐ | ‐ | 16 | 27 | 2 | 1 | Y | Y | Y |
| Liu‐Ambrose 2004 Supervised agility training (87) | P | ‐ | ‐ | ‐ | ‐ | ‐ | ‐ | 25 | 40 | 1 | 3 | N | N | N |
| Liu‐Ambrose 2008 Individual Otago Exercise Program (88) | P | S | ‐ | ‐ | S | ‐ | ‐ | 26 | 65 | 3 | None | Y | Y | Y |
| Lord 1995 Group‐based balance, strength, gait training (91) | P | S | S | ‐ | ‐ | ‐ | ‐ | 52 | 88 | 1 | NR | N | N | N |
| Lord 2003 Group‐based balance, strength, gait training (92) | P | S | S | ‐ | ‐ | ‐ | ‐ | 52 | 96 | 1 | NR | N | N | N |
| Luukinen 2007 Individual balance and gait training (94) | P | ‐ | ‐ | ‐ | S | ‐ | ‐ | 70 | 161 | 3 | None | Y | N | Y |
| Madureira 2007 Group‐based balance training and walking plus home practice (96) | P | ‐ | ‐ | ‐ | S | ‐ | ‐ | 40 | 100 | 4 | NR | N | N | N |
| McMurdo 1997 Group‐based balance training (97) | P | ‐ | ‐ | ‐ | ‐ | ‐ | ‐ | 60 | 135 | 1 | NR | N | N | N |
| Miko 2017 Individual, partially supervised balance training (100) | P | ‐ | ‐ | ‐ | ‐ | ‐ | ‐ | 52 | 312 | 4 | NR | N | Y | Y |
| Morgan 2004 Group‐based strength, balance and gait training (102) | P | S | S | ‐ | ‐ | ‐ | ‐ | 8 | 18 | 1 | 5 | Y | Y | Y |
| Nitz 2004 Group‐based balance (106) | P | ‐ | ‐ | ‐ | ‐ | ‐ | ‐ | 10 | 10 | 1 | 6 | N | Y | Y |
| Reinsch 1992 Group‐based balance and strength training (110) | P | S | ‐ | ‐ | ‐ | ‐ | ‐ | 52 | 156 | 1 | NR | N | N | N |
| Robertson 2001 Individual Otago Exercise Program (112) | P | S | ‐ | ‐ | S | ‐ | ‐ | 52 | 96 | 3 | None | Y | Y | Y |
| Sakamoto 2013 One leg stand balance training (114) | P | ‐ | ‐ | ‐ | ‐ | ‐ | ‐ | 26 | 6 | 3 | None | Y | Y | Y |
| Sales 2017 Group‐based strength, balance, co‐ordination, mobility and flexibility (115) | P | S | ‐ | ‐ | ‐ | ‐ | ‐ | 18 | 45 | 1 | 6-8 | Y | Y | Y |
| Siegrist 2016 Group‐based balance, strength, power and gait training plus home practice (118) | P | S | ‐ | ‐ | ‐ | ‐ | ‐ | 16 | 16 | 4 | 4-12 | Y | Y | Y |
| Skelton 2005 Group‐based FaME balance and strength training plus home practice (119) | P | S | ‐ | ‐ | S | ‐ | ‐ | 36 | 62 | 4 | NR | Y | Y | Y |
| Smulders 2010 Group‐based balance and gait training using an obstacle avoidance course (120) | P | ‐ | ‐ | ‐ | S | ‐ | S‐ training in fall techniques, lifting techniques | 5.5 | 21 | 1 | NR | N | N | N |
| Trombetti 2011 Group‐based balance and gait training (124) | P | ‐ | ‐ | ‐ | ‐ | ‐ | ‐ | 26 | 26 | 1 | NR | N | N | N |
| Weerdesteyn 2006 Group‐based balance and gait training using an obstacle avoidance course (130) | P | ‐ | ‐ | ‐ | S | ‐ | ‐ | 5 | 15 | 1 | NR | N | N | N |
| Wolf 1996 Individual, computerised balance training on force platform (131) | P | ‐ | ‐ | ‐ | ‐ | ‐ | ‐ | 15 | 11 | 2 | 1 | Y | Y | Y |
| Yang 2012 Individual Otago Exercise Program (138) | P | S | ‐ | ‐ | S | ‐ | ‐ | 26 | 87 | 3 | None | Y | Y | Y |
| **3D** |  |  |  |  |  |  |  |  |  |  |  |  |  |  |
| Day 2015 Group‐based Tai Chi (48) | ‐ | ‐ | ‐ | P | ‐ | ‐ | ‐ | 48 | 96 | 1 | 12-16 | N | N | N |
| Huang 2010 Group‐based Tai Chi (65) | ‐ | ‐ | ‐ | P | ‐ | ‐ | ‐ | 22 | 40 | 1 | NR | N | N | N |
| Li 2005 Group‐based Tai Chi (82) | ‐ | ‐ | ‐ | P | ‐ | ‐ | ‐ | 26 | 78 | 1 | NR | N | N | N |
| Li 2018 Tai Ji Quan (83) | - | - | - | P | - | - | - | 24 | 48 | 1 | 9-21 | N | N | Y |
| Lipsitz 2019 Group-based Tai Chi (85) | - | - | - | P | - | - | - | 52 | 164 | 1 | NR | N | NR | NR |
| Logghe 2009 Group‐based Tai Chi (90) | ‐ | ‐ | ‐ | P | ‐ | ‐ | ‐ | 13 | 33 | 4 | 7-14 | N | N | N |
| Merom 2016 Group‐based social dancing (99) | ‐ | ‐ | ‐ | P | ‐ | S | ‐ | 52 | 80 | 1 | 13-43 | N | N | N |
| Taylor 2012 Group‐based Tai Chi, 2x/ week (123) | ‐ | ‐ | ‐ | P | ‐ | ‐ | ‐ | 20 | 40 | 1 | 15 | N | N | N |
| Taylor 2012 Group‐based Tai Chi, 1x/ week (123) | ‐ | ‐ | ‐ | P | ‐ | ‐ | ‐ | 20 | 20 | 1 | 15 | N | N | N |
| Voukelatos 2007 Group‐based Tai Chi (128) | ‐ | ‐ | ‐ | P | ‐ | ‐ | ‐ | 16 | 16 | 1 | NR | N | N | N |
| Wolf 1996 Group‐based Tai Chi (131) | ‐ | ‐ | ‐ | P | ‐ | ‐ | ‐ | 15 | 60 | 4 | NR | N | Y | Y |
| Wolf 2003 Group‐based Tai Chi (132) | ‐ | ‐ | ‐ | P | ‐ | ‐ | ‐ | 48 | 120 | 1 | NR | N | N | N |
| Woo 2007 Group‐based Tai Chi (133) | ‐ | ‐ | ‐ | P | ‐ | ‐ | ‐ | 52 | 156 | 1 | NR | N | N | N |
| Wu 2010 Individual, supervised Tai Chi delivered via video conferencing (134) | ‐ | ‐ | ‐ | P | ‐ | ‐ | ‐ | 15 | 45 | 2 | 1 | N | N | N |
| Wu 2010 Group‐based Tai Chi (134) | ‐ | ‐ | ‐ | P | ‐ | ‐ | ‐ | 15 | 45 | 1 | NR | N | N | N |
| Wu 2010 Individual Tai Chi with DVD instruction (134) | ‐ | ‐ | ‐ | P | ‐ | ‐ | ‐ | 15 | 45 | 3 | None | N | N | N |
| **Multiple primary exercise categories** |  |  |  |  |  |  |  |  |  |  |  |  |  |  |
| Ansai 2015 Group‐based balance, strength and aerobic training (25) | P | P | ‐ | ‐ | ‐ | P | ‐ | 16 | 48 | 1 | NR | Y | Y | Y |
| Arkkukangas 2019 Individual Otago Exercise Program + motivational interviewing (20, 139) | P | S | ‐ | ‐ | S | ‐ | P | 52 | 84 | 3 | None | Y | Y | Y |
| Barclay 2018 (28) | P | - | - | - | P | - | - | 9 | 18 | 1 | 3 | Y | Y | Y |
| Beyer 2007 Group‐based balance, strength and flexibility training (32) | P | P | P | ‐ | ‐ | ‐ | ‐ | 26 | 52 | 1 | 5-7 | Y | Y | Y |
| Brown 2002 Group‐based balance, strength and aerobic training (34) | P | P | ‐ | ‐ | ‐ | S | S ‐ co‐ordination activities | 16 | 32 | 1 | NR | Y | Y | Y |
| Buchner 1997 Group‐based strength training (35) (combined with endurance and combined groups in analysis)* | ‐ | P | ‐ | ‐ | ‐ | P | ‐ | 25 | 78 | 1 | NR | Y | Y | Y |
| Bunout 2005 Group‐based balance, strength and walking (36) | P | P | ‐ | ‐ | ‐ | P | ‐ | 52 | 74 | 1 | NR | Y | Y | Y |
| Cerny 1998 Group‐based balance, strength, flexibility, aerobic training and brisk walking (39) | P | P | P | ‐ | ‐ | P | ‐ | 24 | 108 | 1 | NR | NR | NR | NR |
| Clemson 2012 Individual balance and strength training (42) | P | P | ‐ | ‐ | ‐ | ‐ | ‐ | 52 | 78 | 3 | None | Y | Y | Y |
| Gill 2016 Group and home‐based balance, strength, flexibility and walking training (55) | P | P | S | ‐ | P | ‐ | ‐ | 96 | 180 | 4 | NR | Y | Y | Y |
| Haines 2009 Home strength and balance program with DVD/workbook (58) | P | P | ‐ | P | ‐ | ‐ | ‐ | 8 | 48 | 3 | None | Y | N | Y |
| Halvarsson 2016 Group‐based progressive balance training plus walking (60) | P | ‐ | ‐ | ‐ | P | ‐ | ‐ | 12 | 45 | 4 | 6-10 | Y | Y | Y |
| Hauer 2001 Group‐based progressive strength and balance training (62) | P | P | ‐ | ‐ | P | ‐ | ‐ | 12 | 87 | 1 | 4-6 | Y | Y | Y |
| Irez 2011 Group‐based pilates (68) | P | P | ‐ | ‐ | ‐ | ‐ | ‐ | 12 | 36 | 1 | NR | Y | Y | Y |
| Kamide 2009 Individual balance and strength training (70) | P | P | ‐ | ‐ | ‐ | ‐ | ‐ | 26 | 39 | 3 | None | N | N | N |
| Karinkanta 2007 Combined group‐based balance, agility and resistance training (71) | P | P | ‐ | ‐ | ‐ | ‐ | ‐ | 52 | 104 | 1 | NR | Y | Y | Y |
| Kim 2014 Group‐based balance and strength training (74) | P | P | ‐ | ‐ | ‐ | ‐ | ‐ | 52 | 54 | 1 | NR | Y | Y | Y |
| Lehtola 2000 Group‐based balance and flexibility training plus walking and home practice (81) | P | ‐ | P | ‐ | P | ‐ | ‐ | 26 | 78 | 4 | NR | N | N | N |
| Li 2018 Group-based balance, gait, resistance and flexibility training (83) | P | P | P | - | - | - | - | 24 | 48 | 1 | 9-21 | Y | Y | Y |
| Liu-Ambrose 2019 Individualised Otago Exercise Program (89) | P | P | - | - | P | - | - | 52 | 130 | 3 | None | Y | Y | Y |
| Means 2005 Group‐based balance, strength, flexibility, gait training and walking (98) | P | P | P | ‐ | S | ‐ | ‐ | 6 | 27 | 1 | 6-8 | Y | Y | Y |
| Ng 2015 Group‐based strength and balance training plus home practice (19, 105) | P | P | ‐ | ‐ | ‐ | ‐ | ‐ | 12 | 24 | 4 | 8-10 | Y | Y | Y |
| Park 2008 Strength and balance and endurance training (109) | P | S | S | ‐ | ‐ | P | ‐ | 48 | 144 | 1 | NR | N | N | N |
| Rubenstein 2000 Group‐based balance, strength and endurance (113) | S | P | ‐ | ‐ | ‐ | P | ‐ | 12 | 54 | 1 | 8-10 | N | N | N |
| Sherrington 2014 home‐based strength and balance program (116) | P | P | ‐ | ‐ | ‐ | ‐ | ‐ | 52 | 138 | 3 | None | Y | Y | Y |
| Suzuki 2004 Group‐based strength, balance and gait training plus home practice (122) | P | P | P | P | ‐ | ‐ | ‐ | 26 | 49 | 4 | NR | N | N | N |
| Uusi‐Rasi 2015 Group‐based balance and strength training plus home practice (125) | P | P | ‐ | ‐ | ‐ | ‐ | ‐ | 104 | 260 | 4 | NR | Y | Y | Y |
| Vogler 2009 home‐based strength training with weight-bearing, functional tasks (127) | P | P | ‐ | ‐ | ‐ | ‐ | ‐ | 12 | 22 | 3 | None | Y | Y | Y |

^a^ Classification (P = Primary; S = Secondary); ^b^ Delivery mode (1 = Group; 2 = Individual supervised; 3 = Individual unsupervised; 4 = Group + Home exercise); ^c^ Participants per instructor (1 = if delivery mode was individual supervised, None = if delivery mode was individual unsupervised); * An a priori decision was made to combine three intervention arms of Buchner 1997(35) as falls data were not available for individual intervention arm; N = No, Y = Yes, NR = Not reported
