# Supplementary material for: Evidence on physical activity and falls prevention for people aged 65+ years: systematic review to inform the WHO guidelines on physical activity and sedentary behaviour
Source: Int J Behav Nutr Phys Act. 2020 Nov 26;17:144. doi: 10.1186/s12966-020-01041-3 (PMC7689963; doi:10.1186/s12966-020-01041-3)
Supplement: Supplementary file 6 — Additional file 6: Table S4. Components of studies in categories of exercise not found to prevent falls [file 12966_2020_1041_MOESM6_ESM.docx]

Supplementary Table 4: Components of studies in categories of exercise not found to prevent falls (9)

| **First author, year and interventions** | **Type of exercise according to ProFaNE classification ^16^ ^a^** | | | | | | | **Duration of intervention (weeks)** | **Hours of intervention** | **Delivery mode^b^** | **Participants per instructor^c^** | **Tailored to the individual initially** | **Progressed based on individual assessment** | **Tailored in intensity or type** |
| --- | --- | --- | --- | --- | --- | --- | --- | --- | --- | --- | --- | --- | --- | --- |
|  | **Balance or functional training** | **Strength or resistance training** | **Flexibility training** | **3D exercise** | **General physical activity** | **Endurance exercise** | **Other exercise** |  |  |  |  |  |  |  |
| **Strength/resistance (including power)** |  |  |  |  |  |  |  |  |  |  |  |  |  |  |
| Ansai 2015 Group‐based progressive strength training (25) | ‐ | P | ‐ | ‐ | ‐ | ‐ | ‐ | 16 | 48 | 1 | NR | Y | Y | Y |
| Carter 2002 Group‐based Osteofit strength training (38) | S | P | ‐ | ‐ | ‐ | ‐ | ‐ | 20 | 27 | 1 | NR | N | N | N |
| Fiatarone 1997 Individual high‐intensity progressive resistance training (52) | ‐ | P | ‐ | ‐ | ‐ | ‐ | ‐ | 16 | 30 | 3 | None | NR | Y | Y |
| Grahn Kronhed 2009 Group‐based strength and balance training (56) | S | P | S | ‐ | ‐ | S | ‐ | 16 | 32 | 1 | NR | Y | Y | Y |
| Karinkanta 2007 Group‐based resistance training (71) | ‐ | P | ‐ | ‐ | ‐ | ‐ | ‐ | 52 | 104 | 1 | NR | N | Y | Y |
| Latham 2003 Resistance exercise (80) | ‐ | P | ‐ | ‐ | ‐ | ‐ | ‐ | 10 | 16 | 3 | None | Y | Y | Y |
| Liu‐Ambrose 2004 Supervised, high‐intensity resistance training (87) | ‐ | P | ‐ | ‐ | ‐ | ‐ | ‐ | 25 | 40 | 1 | 2 | Y | Y | Y |
| Vogler 2009 home‐based seated lower limb strength exercises (127) | ‐ | P | ‐ | ‐ | ‐ | ‐ | ‐ | 12 | 22 | 3 | None | Y | Y | Y |
| Woo 2007 Group‐based resistance training (133) | S | P | ‐ | ‐ | ‐ | ‐ | ‐ | 52 | 156 | 1 | NR | N | N | N |
| **General Physical activity** |  |  |  |  |  |  |  |  |  |  |  |  |  |  |
| Ebrahim 1997 Brisk walking (50) | ‐ | ‐ | ‐ | ‐ | P | ‐ | ‐ | 104 | 216 | 3 | None | N | Y | Y |
| Resnick 2002 Individual or group‐based walking (111) | ‐ | ‐ | ‐ | ‐ | P | ‐ | ‐ | 26 | 39 | 4 | NR | Y | Y | Y |
| Voukelatos 2015 Individual walking program (129) | ‐ | ‐ | ‐ | ‐ | P | ‐ | ‐ | 48 | 120 | 3 | None | N | N | N |
| **Other** |  |  |  |  |  |  |  |  |  |  |  |  |  |  |
| Oliveira 2019 (108) | - | - | - | - | - | - | Health coaching, pedometer | 26 | 4 | 1 | None | Y | Y | Y |
| **Exercise vs exercise** |  |  |  |  |  |  |  |  |  |  |  |  |  |  |
| Ballard 2004 Group‐based balance, strength and aerobic training for 15 weeks (27) | P | S | ‐ | ‐ | ‐ | S | ‐ | 15 | 45 | 1 | 5 | NR | NR | NR |
| Ballard 2004 Group‐based balance, strength and aerobic training for 2 weeks (27) | P | S | ‐ | ‐ | ‐ | S | ‐ | 2 | 6 | 1 | 5 | NR | NR | NR |
| Barker 2016 Group‐based Pilates focused on balance and strength plus home practice (29) | P | S | ‐ | ‐ | ‐ | ‐ | ‐ | 12 | 54 | 4 | 4-6 | Y | Y | Y |
| Barker 2016 Individual strength and balance (29) | P | S | ‐ | ‐ | ‐ | ‐ | ‐ | 12 | 30 | 3 | None | Y | Y | Y |
| Davis 2011 Group‐based progressive high intensity resistance training once weekly (46) | ‐ | P | ‐ | ‐ | ‐ | ‐ | ‐ | 52 | 52 | 1 | NR | Y | Y | Y |
| Davis 2011 Group‐based progressive high intensity resistance training twice weekly (46) | ‐ | P | ‐ | ‐ | ‐ | ‐ | ‐ | 52 | 104 | 1 | NR | Y | Y | Y |
| Davis 2011 Group‐based balance and tone (46) | P | ‐ | S | ‐ | ‐ | ‐ | ‐ | 52 | 104 | 1 | NR | N | N | N |
| Freiberger 2007 Group‐based psychomotor program (53) | P | P | ‐ | ‐ | ‐ | ‐ | P‐ perceptual training | 16 | 32 | 4 | 7.5 | N | Y | Y |
| Freiberger 2007 Group‐based balance, strength, flexibility, endurance (53) | P | P | P | ‐ | ‐ | P | ‐ | 16 | 32 | 4 | 7.5 | N | Y | Y |
| Helbostad 2004 Combined group and home‐based balance and strength training (63) | P | S | ‐ | ‐ | ‐ | ‐ | ‐ | 12 | 51 | 4 | 5-8 | Y | Y | Y |
| Helbostad 2004 Individual home balance and strength training (63) | P | S | ‐ | ‐ | ‐ | ‐ | ‐ | 12 | 27 | 3 | None | N | N | N |
| Hwang 2016 Individually supervised Tai Chi (66) | ‐ | ‐ | ‐ | P | ‐ | ‐ | ‐ | 24 | 48 | 2 | 1 | Y | Y | Y |
| Hwang 2016 Individually supervised balance and strength training (66) | P | S | S | ‐ | ‐ | ‐ | ‐ | 24 | 24 | 2 | 1 | Y | Y | Y |
| Kemmler 2010 Group‐based balance, gait flexibility and strength training plus home practice (72) | P | P | P | ‐ | ‐ | S | ‐ | 78 | 1086 | 4 | NR | Y | Y | Y |
| Kemmler 2010 Group‐based low intensity, low frequency balance and endurance training (72) | P | ‐ | P | ‐ | ‐ | S | ‐ | 78 | 10 | 1 | NR | N | N | N |
| Kwok 2016 Group‐based balance, strength and aerobic training plus home practice (77) | P | P | ‐ | ‐ | ‐ | P | ‐ | 12 | 24 | 1 | 6-8 | Y | Y | Y |
| Kwok 2016 Balance, strength and aerobic training using the Nintendo WiiActive (77) | P | P | ‐ | ‐ | ‐ | P | ‐ | 12 | 24 | 1 | 6-8 | Y | Y | Y |
| Kyrdalen 2014 Group‐based Otago Exercise Program (78) | P | S | ‐ | ‐ | S | ‐ | ‐ | 12 | 16 | 4 | 4-8 | Y | Y | Y |
| Kyrdalen 2014 Individual Otago Exercise Program (78) | P | S | ‐ | ‐ | S | ‐ | ‐ | 12 | 16 | 3 | None | Y | Y | Y |
| LaStayo 2017 Resisted lower limb exercise in standing and leg press (79) | P | P | S | ‐ | S | ‐ | ‐ | 12 | 36 | 1 | 2-5 | Y | Y | Y |
| LaStayo 2017 Resisted lower limb exercise using recumbent stepper‐ergometer (79) | P | P | S | ‐ | S | ‐ | ‐ | 12 | 36 | 1 | 2-6 | Y | Y | Y |
| Liston 2014 Group‐based modified Otago Exercise Program plus individual, partially supervised multisensory balance training (86) | P | S | ‐ | ‐ | S | ‐ | ‐ | 8 | 28 | 4 | NR | Y | Y | Y |
| Liston 2014 Group‐based modified Otago Exercise Program plus individual, partially supervised flexibility training (86) | P | S | S | ‐ | S | ‐ | ‐ | 8 | 28 | 4 | NR | Y | N | Y |
| Lurie 2013 Standard Physical Therapy program + surface perturbation treadmill training (93) | P | S | S | ‐ | ‐ | ‐ | S‐ slip and trip training | Variable | Variable | 2 | 1 | Y | Y | Y |
| Lurie 2013 Standard Physical Therapy program(93) | P | S | ‐ | ‐ | ‐ | ‐ | ‐ | Variable | Variable | 2 | 1 | Y | Y | Y |
| Mirelman 2016 Individual, supervised treadmill training (101) | P | ‐ | ‐ | ‐ | ‐ | P | ‐ | 6 | 14 | 2 | 1 | Y | Y | Y |
| Mirelman 2016 Individual, supervised treadmill training plus virtual reality (101) | P | ‐ | ‐ | ‐ | ‐ | S | ‐ | 6 | 14 | 2 | 1 | Y | Y | Y |
| Morone 2016 Group‐based balance training using Wii‐Fit (103) | P | ‐ | ‐ | ‐ | S | ‐ | ‐ | 8 | 16 | 1 | NR | N | N | N |
| Morone 2016 Group‐based balance training (103) | S | ‐ | P | ‐ | ‐ | ‐ | ‐ | 8 | 16 | 1 | NR | N | N | N |
| Morrison 2018 Group‐based balance training (104) | P | ‐ | ‐ | ‐ | ‐ | ‐ | ‐ | 12 | 22 | 1 | 3-5 | N | N | N |
| Morrison 2018 Home‐based strength, balance and aerobic Wii Fit program (104) | P | ‐ | ‐ | ‐ | ‐ | S | ‐ | 12 | 22 | 2 | 1 | Y | Y | Y |
| Okubo 2016 Group‐based Tai Chi and Otago Exercise Program plus home practice (107) | S | S | ‐ | P | S | ‐ | ‐ | 64 | 88 | 1 | NR | N | Y | Y |
| Okubo 2016 Group‐based brisk walking (107) | ‐ | ‐ | ‐ | ‐ | P | ‐ | ‐ | 64 | 120 | 1 | NR | Y | Y | Y |
| Shigematsu 2008 Group‐based stepping training on felt mat (117) | P | ‐ | ‐ | ‐ | ‐ | ‐ | ‐ | 12 | 56 | 1 | NR | N | Y | Y |
| Shigematsu 2008 Group‐based walking (117) | P | ‐ | ‐ | ‐ | P | ‐ | ‐ | 12 | 16 | 1 | NR | N | Y | Y |
| Steadman 2003 Standard, individualised physiotherapy focused on functional training plus balance training (121) | P | ‐ | ‐ | ‐ | ‐ | ‐ | ‐ | 6 | 9 | 2 | 1 | Y | Y | Y |
| Steadman 2003 Standard, individualised physiotherapy focused on functional training (121) | P | ‐ | ‐ | ‐ | ‐ | ‐ | ‐ | 4 | 6 | 2 | 1 | Y | N | Y |
| Verrusio 2017 Individual, supervised balance and gait training using exoskeleton human body posturizer (126) | P | ‐ | ‐ | ‐ | ‐ | ‐ | ‐ | 52 | 156 | 2 | 1 | Y | NR | Y |
| Verrusio 2017 Individual, supervised balance and gait training (126) | P | ‐ | ‐ | ‐ | ‐ | ‐ | ‐ | 52 | 157 | 2 | 1 | Y | NR | Y |
| Yamada 2010 Group‐based indoor walking (135) | S | S | S | ‐ | P | ‐ | ‐ | 16 | 24 | 1 | NR | Y | Y | Y |
| Yamada 2010 Group‐based trail walking (135) | P | S | S | ‐ | S | ‐ | ‐ | 16 | 24 | 1 | NR | Y | Y | Y |
| Yamada 2012 Group‐based balance, strength, flexibility and gait training involving complex obstacle course (136) | P | S | S | ‐ | S | ‐ | ‐ | 24 | 24 | 1 | NR | N | N | N |
| Yamada 2012 Group‐based balance, strength, flexibility and gait training involving simple obstacle course (136) | P | S | S | ‐ | S | ‐ | ‐ | 24 | 24 | 1 | NR | N | N | N |
| Yamada 2013 Group‐based balance, strength, flexibility and gait training including stepping mat (137) | P | S | S | ‐ | S | ‐ | ‐ | 24 | 18 | 1 | NR | N | N | N |
| Yamada 2013 Group‐based balance, strength, flexibility and gait training plus indoor walking (137) | P | S | S | ‐ | S | ‐ | ‐ | 24 | 18 | 1 | NR | N | N | N |

^a^ Classification (P = Primary; S = Secondary); ^b^ Delivery mode (1 = Group; 2 = Individual supervised; 3 = Individual unsupervised; 4 = Group + Home exercise);^c^ Participants per instructor (1 if delivery mode was individual supervised, None if delivery mode was individual unsupervised); An a priori decision was made to combine three intervention arms of Buchner 1997(35) as falls data were not available for individual intervention arm; N = No, Y = Yes, NR = Not reported
